# Neural Correlates of Borderline Personality Disorder (BPD) Based on Electroencephalogram (EEG)—A Mechanistic Review

**DOI:** 10.3390/ijms26178230

**Published:** 2025-08-25

**Authors:** James Chmiel, Donata Kurpas

**Affiliations:** 1 Faculty of Physical Culture and Health, Institute of Neurofeedback and tDCS Poland, 70-393 Szczecin, Poland; 2 Department of Family and Pediatric Nursing, Faculty of Health Sciences, Wrocław Medical University, 51-618 Wrocław, Poland

**Keywords:** borderline personality disorder, BPD, electroencephalography, electroencephalogram, EEG, QEEG, neurophysiology, neural correlates, oscillations

## Abstract

Borderline Personality Disorder (BPD) is marked by emotional dysregulation, instability in self-image and relationships, and high impulsivity. While functional magnetic resonance imaging (fMRI) studies have provided valuable insights into the disorder’s neural correlates, electroencephalography (EEG) may capture real-time brain activity changes relevant to BPD’s rapid emotional shifts. This review summarizes findings from studies investigating resting state and task-based EEG in individuals with BPD, highlighting common neurophysiological markers and their clinical implications. A targeted literature search (1980–2025) was conducted across databases, including PubMed, Google Scholar, and Cochrane. The search terms combined “EEG” or “electroencephalography” with “borderline personality disorder” or “BPD”. Clinical trials and case reports published in English were included if they recorded and analyzed EEG activity in BPD. A total of 24 studies met the inclusion criteria. Findings indicate that individuals with BPD often show patterns consistent with chronic hyperarousal (e.g., reduced alpha power and increased slow-wave activity) and difficulties shifting between vigilance states. Studies examining frontal EEG asymmetry reported varying results—some linked left-frontal activity to heightened hostility, while others found correlations between right-frontal shifts and dissociation. Childhood trauma, mentalization deficits, and dissociative symptoms were frequently predicted or correlated with EEG anomalies, underscoring the impact of adverse experiences on neural regulation—however, substantial heterogeneity in methods, small sample sizes, and comorbid conditions limited study comparability. Overall, EEG research supports the notion of altered arousal and emotion regulation circuits in BPD. While no single EEG marker uniformly defines the disorder, patterns such as reduced alpha power, increased theta/delta activity, and shifting frontal asymmetries converge with core BPD features of emotional lability and interpersonal hypersensitivity. More extensive, standardized, and multimodal investigations are needed to establish more reliable EEG biomarkers and elucidate how early trauma and dissociation shape BPD’s neurophysiological profile.

## 1. Introduction

Impulsive actions, ongoing emotional instability, and trouble sustaining stable relationships and self-image are all hallmarks of Borderline Personality Disorder (BPD), a challenging and complicated mental health illness. Since 0.7% to 2.7% of people may experience it at some point in their lives [1], it is a serious problem for both people and society. In community surveys, overall prevalence does not differ meaningfully by sex (e.g., 6.2% women vs. 5.6% men in the U.S. NESARC study) [2]. By contrast, clinical samples skew female (~75%), likely reflecting help-seeking, referral patterns, differences in comorbidity, and symptom expression [3]. Clinically, women with BPD more often present with mood/anxiety and eating disorders and greater affective instability, whereas men with BPD more often show externalizing patterns (substance use and antisocial traits) [4]. Settings also matter—in some forensic/justice samples of men, point prevalence is very high (≈20%). Together, the experts explain why individual studies sometimes report female- or male-predominant rates despite similar community prevalence [5].

Nevertheless, because of biases in professional settings and variations in how symptoms present, BPD is frequently underdiagnosed [6]. Fundamentally, BPD is characterized by an incapacity to control emotions properly [7]. Even minor stressors can precipitate intense episodes of anger, despair, or anxiety in BPD; significantly, the expression and prevalence of these features vary with age. BPD typically emerges in adolescence, with community estimates in youth and young adults (≈16–22 years) around 1.4–3.2% and cumulative prevalence approaching ~3% by early adulthood [8]; during this period, impulsivity, self-injury, and marked affective lability are most prominent [9]. In adults aged 25–40, population estimates usually fall between ~1–6% (method-dependent), with higher rates at the younger end of this band and a gradual decline thereafter. At the same time, good reactivity and interpersonal instability remain impairing, and overt impulsivity tends to attenuate with increasing age [10]. In older adults (≈70–90 years), available epidemiology suggests a lower prevalence (roughly ~2%) [11] and a further reduction in externalizing behaviors, although chronic interpersonal hypersensitivity, fear of abandonment, and dysphoria may persist [10]. Symptom expression can interact with age. For example, younger men had higher scores for identity problems and self-harm than did older men. Younger women had higher scores for identity problems and affective instability than did older women [12]. Desperate attempts to prevent actual or perceived rejection might result from this emotional reactivity, which also feeds the fear of abandonment. It is common for relationships to be turbulent, with a pattern of alternating between appreciating and idealizing other people. Another defining characteristic is impulsivity, which shows itself in actions like substance misuse, careless spending, or self-harm as a way for people to deal with intense emotional suffering [13]. The lives of people with BPD are further complicated by persistent feelings of emptiness and a lack of a constant sense of self.

BPD has its roots in a confluence of environmental variables and genetic vulnerability. Adverse early experiences, such as inconsistent caring, neglect, or abuse, may have a particularly negative impact on children who are genetically predisposed to heightened emotional sensitivity. These encounters have the potential to alter brain development in ways that raise the likelihood of developing BPD [14]. Particularly in the areas of impulse control and mood regulation, neurobiological research has shed important light on the disorder’s underlying causes. Structural and functional brain imaging studies have identified anomalies in several essential brain areas. For instance, people with BPD often have hyperactive amygdalae, which are involved in processing emotions [15]. This increased activity may explain the extreme emotional reactions and trouble settling down after being agitated.

Pharmacologically, several classes can dampen amygdala responses on fMRI, but this has not translated into disorder-specific benefit. In humans, benzodiazepines acutely attenuate amygdala BOLD activity (dose-dependent lorazepam effects) and modulate central-amygdala microcircuits [16], yet in BPD they are generally discouraged (except for very brief crisis use) because of disinhibition and dependence risk [17]. SSRIs tend to reduce amygdala reactivity to negative stimuli after 6–12 weeks (shown across multiple fMRI studies in depression/healthy samples) [18]. Still, they do not improve the core pathology of BPD and are used adjunctively for comorbid depression/anxiety [17]. Some second-generation antipsychotics can alter amygdala–prefrontal connectivity [19], but high-quality evidence shows little to no effect on overall BPD severity [20].

On the other hand, there is less activity in the prefrontal cortex, which aids in controlling emotions and impulses [21]. The emotional and behavioral dysregulation that characterizes BPD is believed to be caused by this imbalance between the amygdala and prefrontal cortex [21]. People with BPD also exhibit decreased volume in other parts of their brains, including the hippocampus [22,23], which is involved with memory [24]. Additionally, there seems to be aberrant activity in the anterior cingulate cortex [25], which is essential for emotional awareness [26] and impulse control [27].

Functional magnetic resonance imaging shows altered brain activity and structure in BPD. However, the oldest and first neuroimaging technique is electroencephalography (EEG). In EEG, electrodes are applied to the scalp to measure the electrical activity produced by neurons in the brain’s cortex [28]. Waveforms of this activity are usually recorded using frequency bands (e.g., alpha, beta, theta, and delta), which might represent various physiological or cognitive states [29]. The signals acquired from post-synaptic potentials in cortical neurons are amplified and recorded for additional analysis, and standardized electrode placement techniques, like the 10–20 system, ensure uniform alignment with anatomical landmarks [28,30]. Because of its great temporal precision (in the range of milliseconds), EEG is a perfect tool for researching brain dynamics in real-time, including the exact timing of neural responses to certain stimuli, which are frequently investigated using event-related potentials (ERPs) [29,31]. Besides its temporal benefits, EEG is non-invasive, reasonably priced, portable, and widely utilized in clinical and research settings, such as diagnosing and tracking neurological illnesses, including epilepsy and sleep disorders [31,32]. Given the difficulty of pinpointing the recorded signals’ precise cortical or subcortical origins, the main drawback is reduced spatial resolution compared to fMRI techniques. Careful data preprocessing is also necessary to avoid artifacts brought on by eye blinks, muscle tension, or movement [30]. Despite this, EEG remains a vital tool for recording rapidly shifting cerebral activity in practical settings, especially in domains where real-time brain function monitoring is essential [33]. EEG can reveal fine-grained, moment-to-moment changes in cortical activity, providing critical insights into BPD’s rapid emotional shift characteristic, even though fMRI has revealed significant structural and functional abnormalities.

This review aims to collect all studies examining EEG activity in BPD. BPD is a complex brain disorder, and it is essential to understand its neural correlates. The neural characterization of BPD derived from fMRI is well established; however, no similar work on EEG has been published to date. This review aims to capture patterns in cortical activity, as it is crucial for developing therapeutic interventions. In addition, the review will analyze the limitations of previous studies and make recommendations for conducting future research. Only studies with raw EEG recording, resting state, or combined with different paradigms were included in the review. Other methodologies, such as ERP, were not included.

## 2. Methods

This review aims to examine EEG activity in patients with BPD. Strict selection criteria and a comprehensive literature search were employed to ensure the findings’ validity and applicability. The strategy, which followed recognized protocols for systematic reviews and evidence synthesis (PRISMA), concentrated on locating clinical trials and case studies that assessed EEG activity in BPD. However, not every element of the PRISMA process was included because this is a mechanistic review rather than a systematic one.

### 2.1. Data Sources and Search Strategy

To write this evaluation, J.Ch. and D.K. used the following combination of terms in an independent, standards-based internet search: “EEG” OR “electroencephalogram” OR “electroencephalography” OR “QEEG” OR “electrophysiology” OR “neurophysiology” AND “borderline personality disorder” OR “borderline” OR “BPD”. In January 2025, a thorough search was carried out across multiple databases, including PubMed/Medline, Research Gate, Google Scholar, and Cochrane, with an emphasis on articles from January 1980 to January 2025. We limited the search to January 1980 onward because (i) BPD entered the official diagnostic nomenclature in DSM-III (1980), improving case-definition consistency and (ii) the late 1970s–1980s saw the transition to digital/computerized EEG and routine quantitative analyses, which substantially improved data quality and comparability [34,35,36].

### 2.2. Study Selection Criteria

Publications must have been case studies or clinical trials published in English between January 1980 and January 2025 to be eligible for inclusion. Papers written in languages other than English were not included.

### 2.3. Screening Process

Various screening techniques were implemented to guarantee that pertinent research was included and studies that did not fit predetermined criteria were eliminated. Two independent reviewers, J.Ch. and D.K., thoroughly examined abstracts and titles in the first screening step.

#### 2.3.1. Title and Abstract Screening

To find studies that fit the inclusion requirements, each reviewer independently evaluated the abstracts and titles of the records that were accessible. The EEG in BPD was the primary focus of the screening criteria.

#### 2.3.2. Full-Text Assessment

After titles and abstracts were first screened, the chosen papers underwent a thorough full-text assessment. To verify that each publication satisfied the eligibility conditions, the reviewers carefully reviewed each one, paying close attention to whether the studies were case studies or clinical trials published in English between January 1980 and January 2025. Studies that did not contain information on EEG results were excluded.

### 2.4. EEG Frequency Bands

We used conventional clinical EEG bands: delta (0.5–4 Hz), theta (4–7 Hz), alpha (8–12 Hz), beta (13–30 Hz), and gamma (~30–80 Hz). Boundaries vary slightly across laboratories but fall within these ranges in standard references [37,38].

## 3. Results

**Figure 1:**
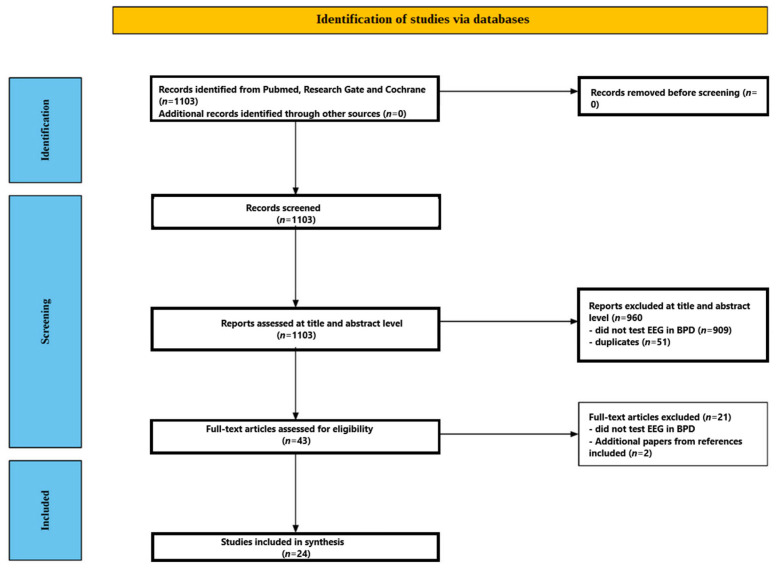
Flow chart depicting the different phases of the systematic review.

Figure 1 illustrates the screening process. Initially, 1103 studies were found through database searches. In total, 960 papers were eliminated after the titles and abstracts were examined; 909 of these studies did not look into EEG in BPD, and 51 were duplicates. A comprehensive full-text analysis was performed on the remaining forty-three manuscripts. Twenty-one publications were later rejected because they failed to look at the EEG in BPD. More specifically, of the matching studies, three were excluded because they used event-related potentials, one study was excluded because it did not report detailed results, one study was excluded because it was in a language other than English, and three studies were excluded because they tested the effects of various substances on the EEG in people with BPD. After carefully examining their substance, 22 papers were determined to satisfy the inclusion requirements. Two more pertinent papers were found by looking through the bibliographies of research that met the review criteria. As a result, the review contained 24 studies in total. From 1998 to 2023, these 24 studies were published [39,40,41,42,43,44,45,46,47,48,49,50,51,52,53,54,55,56,57,58,59,60,61,62]. The included studies are presented in Table 1.

### 3.1. Participants’ Characteristics

Across studies, samples ranged from small, tightly controlled cohorts to larger archival datasets, spanning adolescence through middle adulthood. Most investigations contrasted individuals with BPD against healthy controls (HC), with additional clinical comparators included in several designs—major depressive disorder (MDD) or unipolar depression (UP) [39,45,55,61], bipolar disorder or bipolar II (BD/BD II) [46,54], obsessive–compulsive disorder (OCD) [52], and dysthymia [43]. Special subgroups were also examined, such as self-injurious BPD patients who did versus did not report pain during self-injury [31], and a student sample identified via screening [51]. Sample sizes varied widely: tiny case–control series (e.g., 10 BPD vs. 10 HC) [56] and narrowly selected cohorts [51] contrasted with larger datasets, including a 111-participant qEEG comparison across BPD, BD, and HC [46] and a 146-recording machine-learning dataset spanning MDD and BPD with comorbid depression [61]. Ages typically clustered in the 20s–30s for adult cohorts (e.g., mean ~26–33 years in many studies) [40,41,42,44,45,46,47,48,49,54,55,56,57,58,59], with adolescent and young adult samples specifically targeted in two studies [53,62]. Sex distributions were frequently skewed toward women—several cohorts were female-only [39,42,45,47,53,60]—although mixed-sex samples were also represented [40,44,46,48,49,51,52,54,56,59,61], and one inpatient cohort was male-only [43]. Handedness was controlled in some designs (e.g., right-handed females only in [39]; matched for handedness in [41]).

Diagnostic ascertainment primarily relied on contemporary nosology and structured interviews (e.g., SCID with DSM-IV/5 criteria) to establish BPD diagnoses and to screen HCs for psychiatric/neurological conditions [40,41,42,43,44,46,48,49,50,51,52,53,54,56,59,60,61,62]. Earlier clinical EEG studies used DSM-III, the Diagnostic Interview for Borderlines (DIB), or clinical judgment when formal criteria were not yet standardized [43,47,55,58]. Comorbidity management and inclusion thresholds varied: some studies imposed stringent exclusions for Axis I/II disorders and neurological disease [43,50], whereas others adopted more naturalistic samples or explicitly included comorbid presentations (e.g., BPD with depression or anxiety in adolescents) [62]. Recruitment settings ranged from inpatient units—often with medication washouts or blinded pharmacologic protocols [43,57,58]—to outpatient and community samples and university settings [51]. Medication status also differed across studies, from fully unmedicated cohorts [48,52] to partially medicated samples with targeted exclusions (e.g., benzodiazepines disallowed but other psychotropics permitted to preserve ecological validity) [41], and designs incorporating washout periods before baseline EEG acquisition [57,58]. The literature is characterized by heterogeneity in age and sex composition, clinical comparators, comorbidity control, and medication exposure—factors that should be considered when interpreting cross-study convergence in EEG findings.

### 3.2. EEG Paradigms and Tasks

Across studies, EEG paradigms ranged from conventional resting state recordings to affective, cognitive, and sensory-challenge tasks. Resting state designs predominated, typically using short eyes-closed (EC) and/or eyes-open (EO) blocks of 3–5 min [40,42,44,51,52,53,54,62], with some extended sessions of 7–20 min to stabilize spectral estimates or enable vigilance staging [46,48,54]. Several protocols alternated EC/EO within a session to probe state-dependent changes [39,40,54,62]. Clinical-style recordings incorporated standardized activation procedures—hyperventilation, photic stimulation, and eye-opening/closing cycles—and, in one study, a subsequent sleep segment to increase the yield of abnormalities [43,47,50,55,57,58,61]—task-based paradigms targeted core BPD constructs. Social rejection was elicited with Cyberball, embedding inclusion, partial exclusion, and complete exclusion phases, with baseline and post-task resting EEG to capture tonic shifts in approach–withdrawal tendencies [39]; a related adolescent design paired FAA measurement with Cyberball to examine moderating effects on rejection sensitivity [62]. Affective provocation included mood induction via IAPS images with pre/post resting state recordings [41] and an emotion regulation task contrasting cognitive reappraisal versus maintenance for hostile/neutral images [60]. Reward/feedback processing was examined using two-choice gambling or guessing tasks that balanced gains and losses and supported time–frequency analyses of feedback-locked oscillations; one study acquired EEG simultaneously with fMRI to link theta dynamics to frontocingulate circuitry [49,53,59]. Sensory-challenge work employed a cold pressor test (CPT) to probe pain processing and dissociation while recording EC EEG throughout baseline and immersion, with repeated pain ratings to align physiology with experience [45]. Vigilance-regulation studies leveraged longer EC resting recordings analyzed with computerized staging (e.g., VIGALL) to quantify arousal levels and transitions, either in BPD versus healthy controls or in cross-diagnostic contrasts (e.g., BPD vs. OCD) [48,52]. Finally, classification-oriented studies standardized brief EC/EO resting EEG (with or without clinical activations) to extract features for machine learning aimed at distinguishing BPD from BD II or MDD with comparable depressive symptomatology [54,61]. Collectively, these paradigms span tonic (resting/vigilance) and phasic (social, reward, pain, and reappraisal) probes, allowing triangulation of arousal regulation, approach–withdrawal motivation, feedback sensitivity, and emotion regulation within the BPD phenotype.

### 3.3. Recording Setups and Durations

EEG acquisition ranged from low-density clinical systems to high-density research caps, with corresponding montage and session length variability. Clinical-era and hospital-based studies commonly used 16–21 channels placed according to the 10–20 system [45,51,55,58], with one longitudinal protocol using 17 channels [57]. Several contemporary resting state and task designs employed 32-channel caps [41,42], while feedback-learning work typically used 64 channels [49]. High-density arrays were adopted for microstate analysis (256 channels) and adolescent FAA work (128-electrode HydroCel nets) to capture fine-grained spatial patterns [44,62]. Most studies positioned electrodes in standard 10–20 locations; a subset explicitly emphasized frontal pairs for asymmetry indices across multiple homologous sites [39,41,42,62]. Artifact handling ranged from clinical visual inspection to modern pipelines with independent component analysis for ocular/motion correction, especially in task-based and multimodal designs [49,54,59].

Durations reflected the study aims. Brief resting state segments of 3–5 min (eyes closed and/or eyes open) were most common [40,42,44,51,52,53,54,62], with several protocols alternating EC/EO blocks within a session to probe state dependence [39,40,54,62]. Longer single-condition rests were used to stabilize spectral estimates or stage vigilance (e.g., 7 min EC in qEEG comparisons, 10 min split EO/EC in classification studies, and 20 min EC for vigilance regulation) [46,48,54]. Task paradigms layered short resting baselines with post-manipulation recordings (e.g., 8 min alternating baseline plus 2 min post-Cyberball in [39]; two 8 min rests bracketing IAPS mood induction in [41]) or embedded continuous EEG during performance (gambling/guessing tasks; reappraisal; cold pressor) with typical single runs lasting a few minutes [45,49,53,59,60]. Clinical protocols were markedly longer and included activation procedures—eye opening/closing, hyperventilation, and photic stimulation—and, in one case, an added sleep segment: 30 min resting plus 30 min sleep [43]; extended 40 min sessions with activations [50]; and multi-time point recordings across a 32-day randomized treatment phase [57]. Likewise, several diagnostic-era studies incorporated hyperventilation and photic stimulation in routine 16-channel recordings [47,55,58,61]. Collectively, setups and durations spanned brief, tightly controlled research blocks to prolonged clinical recordings with activations, a heterogeneity that bears on the comparability of spectral estimates, vigilance state, and the yield of qualitative abnormalities across studies.

### 3.4. EEG Measures and Frequency Bands

Across studies, signal quantification ranged from classical spectral summaries to task-locked, time–frequency dynamics, with a recurrent emphasis on alpha-band indices of frontal asymmetry and broad-band power as putative markers of arousal and affective style. Frontal EEG/alpha asymmetry (FEA/FAA) was typically computed as the natural log–transformed alpha power difference between homologous frontal sites, most often defined as ln(Right)—ln(Left), with alpha treated as inversely related to cortical activation; positive scores therefore indexed relatively greater left-frontal activity (approach motivation), whereas negative scores indexed greater right-frontal activity (withdrawal) [39,41,42,62]. Implementations ranged from dense sets of frontal pairs (e.g., 11 homologous pairs) during baseline and post–social rejection phases [39] to targeted pairs (e.g., F3–F4) with parietal asymmetry included as a control region [62]. Beyond asymmetry, resting-state studies estimated power spectral density (PSD) under eyes-closed and/or eyes-open conditions, covering canonical bands—delta, theta, alpha, beta, and gamma—with several reports subdividing alpha (α1/α2) and beta (β1/β2/β3/high-β) and extending into high-frequency gamma partitions (γ1/γ2/high-γ) for finer granularity [40,44,46,51,54,61]. Statistical testing frequently used nonparametric approaches tailored to EEG (e.g., permutation *t*-tests and cluster-based corrections), and associations with clinical measures were probed via rank-based correlations with multiple-comparison control [40].

Task-based work characterized induced oscillatory responses using wavelet or related time–frequency decompositions. Feedback/gambling and guessing paradigms focused on alpha and low-beta modulations around outcome delivery (e.g., 10 Hz alpha, 15 Hz low-beta) [49] and, in adolescent samples, on theta and delta dynamics of feedback processing [53]. A simultaneous EEG–fMRI study specified theta extraction with a wavelet layer centered at 5.1 Hz (≈4.4–5.8 Hz) and high-beta centered at 25.5 Hz (≈22–29.1 Hz), quantifying peak power in predefined post-stimulus windows (theta: 200–900 ms; high-β: 100–500 ms) to link oscillatory markers to frontocingulate BOLD activity and impulsivity traits [59]. Emotion regulation (reappraisal vs. maintenance) analyses emphasized frontal theta (≈3.5–8.5 Hz), complemented by source- and connectivity-level metrics (e.g., eLORETA, multivariate interaction measures) to index network engagement during cognitive control of affect [60]. Sensory-challenge designs (cold pressor) summarized absolute band-limited power across delta (≈0.75–3 Hz), theta (4–7 Hz), alpha (8–13 Hz), and segmented beta ranges during baseline and nociceptive stimulation to relate oscillatory shifts to dissociation and pain reports [45].

Connectivity and temporal organization were interrogated using coherence and microstate frameworks. Coherence analyses estimated interregional coupling across delta through gamma bands at rest [51,56]. In contrast, high-density recordings supported microstate segmentation alongside concurrent band-limited power summaries (delta 2–4 Hz, theta 4–8 Hz, alpha 8–12 Hz, beta 13–30 Hz) to characterize spatiotemporal stability in intrinsic activity [44]. Arousal regulation was operationalized with EEG-vigilance staging, classifying second-by-second states from high alertness to sleep onset (VIGALL 2.0; stages 0–C) or via A-substage taxonomies (A1–A3, B), enabling quantification of vigilance levels and transitions as trait-like markers of dysregulated arousal in BPD [48,52]. In contrast, earlier and clinically oriented investigations relied on qualitative EEG endpoints—background rhythm features, focal or diffuse slowing (theta/delta), mixed fast–slow activity, paroxysmal or spike–wave patterns, and their localization/lateralization—sometimes across multiple sessions or treatment time points (e.g., carbamazepine vs. placebo) and often in the context of activation procedures (hyperventilation, photic stimulation) to increase diagnostic yield [43,47,50,55,57,58]. Collectively, these measurement strategies span tonic (PSD, coherence, vigilance states, asymmetry) and phasic (time–frequency responses to social, reward, pain, and regulation challenges) characterizations, providing complementary windows on motivational asymmetry, arousal control, large-scale coupling, and feedback-sensitive oscillatory dynamics within the BPD phenotype.

### 3.5. Clinical and Psychological Measures

Across studies, clinical phenotyping combined disorder-level interviews with dimensional self-reports indexing mood, affective lability, dissociation, impulsivity, trauma, and emotion-regulation capacity. Depressive symptom burden was commonly quantified with the Beck Depression Inventory (BDI/BDI-II) and, in one case, the Montgomery–Åsberg Depression Rating Scale (MADRS) [39,41,42,44,45]. General psychopathology was captured with the SCL-90-R, while affective style and state affect were indexed with instruments such as the PANAS-X and aggression/hostility checklists, including rejection-sensitivity measures surrounding social exclusion tasks [39,42]. Emotion-regulation constructs were assessed with the Emotion Regulation Questionnaire (ERQ) and trial-wise ratings during reappraisal paradigms [60]. In contrast, impulsivity was evaluated with the Barratt Impulsiveness Scale (BIS), particularly in feedback-learning and multimodal EEG–fMRI designs [59]. Developmental risk and social-cognitive capacities were addressed via childhood-trauma inventories—most often the Childhood Trauma Questionnaire (CTQ) or the PROVE-ACE—and mentalization measures (PROVE-MC) encompassing emotional awareness and expression [40,41]. Dissociative phenomena were captured with the Dissociative Experiences Scale (DES), especially in pain-challenge work, differentiating self-injuring BPD subgroups by pain reporting; anxiety was measured with the Sheehan Patient-Rated Anxiety Scale, and continuous pain ratings were recorded during the cold pressor test to align physiology with experience [45]. Trait alexithymia—relevant to interoceptive and affect-identification deficits—was indexed using the TAS/TAS-20 [41,42]. Sleep- and arousal-related complaints were collected via subjective sleepiness and prior-night sleep-quality reports alongside vigilance staging to contextualize resting EEG [48]. Cognitive performance entered primarily in classification studies, which paired resting EEG features with executive measures from the Wisconsin Card Sorting Test (WCST) and the Integrated Cognitive Assessment (ICA) to aid discrimination of BPD from BD II [54]; student screening for a high-risk subset was conducted with the MCMI-III before EEG acquisition [51]. Diagnostic ascertainment and control screening typically relied on structured interviews aligned with DSM criteria (e.g., SCID; DIB in earlier work), while several historical clinical EEG studies also compiled symptom lists overlapping with complex partial seizure or episodic dyscontrol syndromes to interpret qualitative EEG findings in context [43,47,55,58]. Collectively, this battery of measures supports linkage of oscillatory markers to specific symptom dimensions—depressed mood, affective instability, dissociation, impulsivity, trauma burden, and emotion-regulation success—rather than to diagnosis alone.

### 3.6. Comparators and Classification Work

Comparative designs were leveraged to test diagnostic specificity beyond the standard BPD–healthy control contrast. Depression comparators were common: several studies juxtaposed BPD with major depressive disorder (MDD) or unipolar depression (UP) to parse effects attributable to mood disturbance versus borderline pathology, including social rejection/approach–withdrawal paradigms [39], vigilance or clinical EEG protocols [52,55], and feature-based discrimination efforts [61]. The BPD–bipolar spectrum boundary was interrogated with both quantitative EEG (qEEG) group comparisons across an extensive frequency range [46] and a focused BPD versus bipolar II (BD II) classification design [54], reflecting the nosological debate over overlap in affective instability and impulsivity. Additional comparators targeted arousal and temperament: an OCD cohort provided a non-mood clinical benchmark for vigilance regulation [52], while a dysthymia inpatient group served as a mood-disordered comparison in a male-only clinical EEG sample [43]. Within-disorder contrasts further refine the phenotype, separating self-injurious BPD subgroups by pain reporting to relate oscillatory dynamics to dissociation and nociception [45] and identifying a screened student subset meeting BPD criteria to examine resting spectra and connectivity in a young, nonclinical recruitment base [51].

Two studies explicitly pursued classification rather than solely group-level inference. Using 10 min of standardized resting EEG split between eyes-closed and eyes-open across 21 channels, plus executive measures (WCST, ICA), one study trained traditional machine-learning models (kNN, SVM, decision trees) to distinguish BPD from BD II, extracting statistical, spectral, and wavelet features from delta through beta bands [54]. A separate clinical EEG dataset—19-channel recordings acquired at rest and during photic stimulation and hyperventilation—applied feature engineering across linear and non-linear domains and evaluated Random Forests and SVM to differentiate primary MDD from BPD with comorbid depression, directly addressing whether “borderline depression” is electrophysiologically distinct from MDD [61]. Although both projects reported feasibility with cross-validated pipelines, they also inherited common limitations of modest sample sizes, heterogeneous preprocessing, and potential confounding by eye state and activation procedures, underscoring the need for harmonized acquisition and transparent model validation.

Beyond explicit classifiers, several comparator frameworks imply discriminative potential. qEEG spectra spanning α1/α2 and multiple β and γ partitions differentiated BPD from BD and HC at rest [46]; vigilance-staging profiles (VIGALL; A- and B-states) distinguished BPD from HC and OCD on arousal dynamics [48,52]; task-locked oscillations during reward feedback (theta/alpha/low-β) and simultaneous EEG–fMRI couplings in frontocingulate circuits highlighted feedback processing as a candidate marker distinct from general depressive load [49,53,59]; and frontal alpha asymmetry during social exclusion or at rest captured approach–withdrawal biases relevant for differentiating BPD from MDD and for stratifying adolescents by borderline features [39,62]. Connectivity (coherence) and microstates offered additional separation at the network level in small samples [44,51,56]. Taken together, comparator-based contrasts and early classification attempts converge on a set of potentially discriminative EEG domains—arousal/vigilance regulation, frontal alpha asymmetry, feedback-related theta/alpha dynamics, and resting connectivity—while also highlighting the field’s methodological heterogeneity (channel density, eye state, activations, medication status, and sex/age composition) that must be addressed before robust diagnostic tools can be realized.

### 3.7. EEG Outcomes in BPD

**Figure 2:**
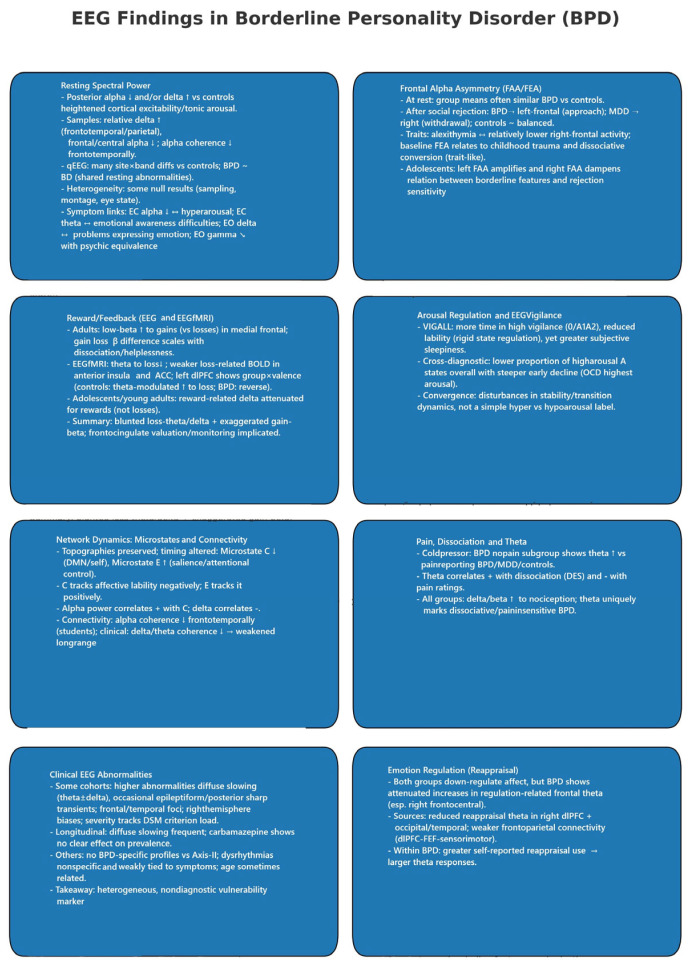
EEG findings in Borderline Personality Disorder.

A summary of EEG findings in BPD is presented in Figure 2.

#### 3.7.1. Resting State Spectral Power

Across resting paradigms, broad-band alterations were more evident when comparing clinical groups to healthy controls than in head-to-head BPD contrasts. Several datasets reported posterior alpha reductions and/or delta increases in BPD, consistent with heightened cortical excitability and tonic arousal [44,51]. Student and small clinical samples converged on elevated relative delta in frontotemporal/parietal sites with reduced frontal/central alpha and diminished alpha coherence frontotemporally [51]. In a larger qEEG survey spanning delta through high gamma, numerous site–band differences separated both BPD and BD from controls, but BPD and BD were statistically indistinguishable on post hoc tests, suggesting partially shared resting abnormalities across affective–impulsive spectra [46]. Not all studies replicated strong spectral group effects; some found no global band differences after conservative controls, emphasizing heterogeneity in sampling, montage, and eye state [44]. At the symptom level, resting-band power related to socio-cognitive features in a trauma/mentalization cohort: lower EC alpha in BPD indexed hyperarousal; EC theta correlated with emotional awareness difficulties; EO delta with problems in emotional expression; and EO gamma was inversely related to psychic equivalence (a marker of impaired mentalization) [40].

#### 3.7.2. Frontal Alpha Asymmetry (FAA/FEA) and Approach–Withdrawal Tendencies

Group means in FAA/FEA were often similar across BPD and controls at rest [39,41,42], yet asymmetry carried explanatory value once context and traits were considered. Following social rejection, BPD shifted toward left-frontal asymmetry (approach motivation), MDD toward right-frontal (withdrawal), and controls remained balanced—mirroring group-specific motivational styles [39]. FAA also tracked individual differences: in BPD, greater alexithymia—especially difficulties describing feelings—associated with relatively lower right-frontal activity [42]; baseline FEA related to childhood trauma and dissociative conversion symptoms, accounting for substantial variance in the BPD group and supporting a trait-like interpretation [41]. Developmentally, FAA moderated the link between borderline features and rejection sensitivity in adolescents: greater left FAA amplified context—high BPD features predicted the highest rejection sensitivity, whereas low features predicted the lowest; right FAA dampened this gradient [62].

#### 3.7.3. Reward/Feedback Processing (Time–Frequency EEG and EEG–fMRI)

Feedback-locked oscillations showed consistent but band-specific alterations. In adults, BPD exhibited enhanced low-beta power to gains (vs. losses) localized to medial frontal regions, and the gain–loss beta difference scaled with symptom severity (e.g., dissociation, helplessness) [49]. In simultaneous EEG–fMRI, BPD showed reduced theta power to loss feedback and weaker feedback-related BOLD in the anterior insula and ACC; theta-informed analyses revealed a group-by-valence interaction in the left dlPFC (controls: stronger theta-modulated dlPFC to loss; BPD: the reverse), linking abnormal frontal theta dynamics to altered cognitive control during aversive outcomes [59]. In adolescents/young adults, reward-related delta power was attenuated specifically for rewards (not losses) in BPD, indicating early-emerging deficits in reward responsiveness [53]. These results point to atypical valuation/monitoring signals: blunted loss-theta/delta and exaggerated gain-beta signatures, with frontocingulate circuitry implicated.

#### 3.7.4. Arousal Regulation and EEG-Vigilance

Vigilance findings indicate dysregulated arousal but vary by method and comparator. Using VIGALL staging over 20 min, BPD spent more time in higher vigilance (stage 0/A1–A2), showed reduced lability (rigid state regulation), yet paradoxically reported greater subjective sleepiness—suggesting a mismatch between perceived and physiological arousal [48]. In a cross-diagnostic framework with OCD and controls, BPD displayed a lower proportion of high-arousal “A” states overall and a steeper early decline. In contrast, OCD maintained the highest arousal—underscoring disorder-specific arousal profiles and the influence of staging taxonomies and time windows on results [52]. Both studies converge on disturbed stability/transition dynamics rather than a simple “hyper-vs-hypoarousal” label.

#### 3.7.5. Network Dynamics: Microstates and Connectivity

High-density microstate analysis showed preserved scalp topographies but altered temporal dynamics: BPD exhibited reduced occurrence/duration/coverage of Microstate C (often associated with DMN/self-referential processing) and increased Microstate E (linked to salience/attentional control). Microstate C negatively tracked affective lability, while Microstate E positively tracked it; alpha power correlated positively with Microstate C and negatively with delta, tying large-scale state organization to arousal and emotional instability [44]. Resting connectivity measures further suggested network inefficiency: alpha coherence was reduced frontotemporally in a student sample [51], and clinical recordings showed lower delta/theta coherence without robust alpha/beta differences, pointing to weakened long-range integration in low-frequency bands relevant to affect and interoception [56].

#### 3.7.6. Pain, Dissociation, and Theta Dynamics

During cold pressor testing, the BPD subgroup reporting no pain during self-injury displayed uniquely elevated theta power relative to pain-reporting BPD, MDD, and controls; theta increases correlated positively with dissociation (DES) and negatively with pain ratings. All groups showed expected delta/beta increases to nociception, but theta distinguished dissociative/pain-insensitive BPD, implicating midline–limbic systems in altered pain awareness and defensive detachment [45].

#### 3.7.7. Clinical EEG Abnormalities and Activation Protocols

Clinical-style EEGs yielded mixed conclusions. Several reports found higher rates of abnormalities in BPD—most commonly diffuse slowing (theta ± delta) and, in some cohorts, epileptiform or posterior sharp transients—with frontal/temporal foci and right-hemisphere biases, and severity scaling with diagnostic load (more DSM criteria → more abnormalities) [43,50,55]. Longitudinal data showed frequent diffuse slowing at multiple time points, with carbamazepine exerting no apparent effect on EEG prevalence [57]. Other studies observed no BPD-specific abnormality profiles or no significant differences versus non-BPD Axis-II controls; when present, dysrhythmias were nonspecific and weakly tied to symptom clusters [47,58]. Age is occasionally related to abnormality burden [43]. Overall, qualitative abnormalities likely index heterogeneous, non-diagnostic neurophysiological vulnerability.

#### 3.7.8. Emotion Regulation (Reappraisal) and Frontal Theta/Connectivity

Both groups successfully down-regulated negative affect during cognitive reappraisal, but BPD showed attenuated increases in regulation-related frontal theta, especially over right frontocentral sensors. Source estimates highlighted reduced reappraisal-theta in the right dlPFC (and occipital/temporal regions), and frontoparietal connectivity increases (e.g., dlPFC–FEF–sensorimotor hubs) were weaker than in controls. Within BPD, better self-reported reappraisal use predicted larger theta responses, linking individual strategy capacity to neural control signals [60].

#### 3.7.9. Diagnostic Differentiation and Machine Learning

QEEG features robustly separated BPD and BD from healthy controls but did not cleanly dissociate BPD from BD at the group level [46]. In contrast, a feature-engineering/machine-learning approach using wavelet and spectral metrics (20-channel, EC/EO) achieved high cross-validated accuracy distinguishing BPD from BD II, with EEG features far outperforming cognitive measures; k-nearest neighbors performed best in that dataset [54]. When the question shifted to MDD versus MDD+BPD, multiple algorithms failed to discriminate groups from routine clinical EEG (including activation procedures), suggesting substantial electrophysiological overlap in depressive presentations regardless of borderline comorbidity [61]. Taken together, discriminability appears task- and comparator-dependent, strongest against healthy controls and mixed versus bipolar comparators, and weakest within depressive spectra.

### 3.8. Quality of Evidence and Risk of Bias

The quality of evidence and risk of bias assessment in the included studies is presented in Table 2.

## 4. Discussion

The studies reviewed offer a multifaceted view of neurophysiological alterations associated with Borderline Personality Disorder. Although they differ widely in methodology—ranging from qualitative EEG assessments to advanced quantitative analyses, microstate evaluations, and simultaneous EEG-fMRI—they converge on several key points. Collectively, these findings underscore that BPD is linked to distinctive neural profiles suggestive of hyperarousal, difficulties with emotion regulation, and, for some individuals, developmental trauma sequelae. However, consistent and disorder-specific EEG markers remain elusive, given the heterogeneity of results and the considerable overlap with other disorders.

### 4.1. Frontal EEG Asymmetry and Approach/Withdrawal Tendencies

Several of the reviewed studies focus on frontal EEG alpha asymmetry (FEA) as a window into the approach (left frontal) versus withdrawal (right frontal) motivational systems [63], both of which are highly relevant to BPD’s clinical picture [64]. In these studies, alpha power is inversely related to cortical activity, meaning lower alpha (i.e., relative “desynchronization”) implies stronger underlying activation. Consequently, left-frontal alpha asymmetry translates into greater right-frontal alpha power (i.e., heightened left-frontal cortical activity). It is typically associated with approach motivation, whereas right-frontal alpha asymmetry indicates greater left-frontal alpha power (i.e., heightened right-frontal cortical activity) linked to withdrawal tendencies [63]. In a study [39], which used the Cyberball task to experimentally induce social rejection, participants with BPD showed a significant shift toward left-frontal asymmetry following the task. At the same time, those with Major Depressive Disorder (MDD) exhibited marked right-frontal asymmetry, reflecting withdrawal. Healthy controls maintained a more balanced profile, suggesting a moderate approach orientation. Notably, the BPD group’s leftward asymmetry correlated with higher self-reported hostility, indicating that increased approach motivation may translate into heightened aggressiveness when individuals perceive a social threat.

In contrast to [39], study [41] reported no significant group-level differences in resting FEA between BPD and controls. However, within the BPD group, baseline frontal asymmetry was strongly predicted by childhood trauma and dissociative symptoms—particularly conversion-type experiences—explaining more than half of the variance. After a pessimistic mood induction, both BPD and control participants shifted toward a right-frontal pattern, suggesting that acute negative affect can transiently prompt a withdrawal-related neural response across individuals; nonetheless, the robust link between trauma, dissociation, and FEA at baseline in BPD highlights how deeply ingrained early adverse experiences may shape frontal-lobe functioning, independent of short-term emotional states. Similarly, a study [42] found no overall group difference in baseline FEA between BPD and controls. Yet, within the BPD group, higher Toronto Alexithymia Scale (TAS-20) scores were tied to relatively lower right-frontal cortical activity (i.e., less right-sided alpha suppression), indicating a weaker withdrawal response and potentially reflecting a blunted attunement to internal emotional cues. By contrast, this link between FEA and alexithymia was absent in healthy participants, suggesting a trait-level marker of emotion-processing difficulties that is more specific to BPD. Overall, these findings indicate that while BPD does not invariably present a single resting state asymmetry pattern, contextual factors (such as social rejection or mood induction) and person-specific variables (such as childhood trauma, dissociation, and alexithymia) significantly modulate alpha asymmetry. For some individuals with BPD, a tendency toward left-frontal dominance may predispose them to hostile or confrontational behavior when threatened.

In contrast, others demonstrate transient or baseline shifts toward right-frontal activity linked to withdrawal, particularly in the presence of substantial trauma histories or dissociative traits. Consequently, FEA in BPD appears highly dynamic and individualized rather than a uniform diagnostic biomarker, yet it still highlights the broader dysregulation of approach/withdrawal systems in this disorder. Beyond frontal asymmetry and approach-withdrawal tendencies, EEG findings reveal additional neuronal mechanisms underlying BPD, including dysregulated limbic-prefrontal connectivity, impaired cognitive control, and disturbances in arousal regulation [41,46,61].

Additionally, a study [62], which also used the Cyberball paradigm in an adolescent sample, demonstrated that frontal FAA moderated the relationship between BPD symptoms and feelings of social rejection. Specifically, hierarchical regression analyses revealed that adolescents with greater left FAA and high BPD symptom severity exhibited the highest levels of rejection sensitivity. In contrast, those with greater left FAA but low BPD symptoms showed the lowest rejection sensitivity. Conversely, adolescents with better right FAA displayed moderate and consistent levels of rejection sensitivity, regardless of BPD severity. These findings suggest that left FAA may function as a differential susceptibility factor—amplifying either positive or negative outcomes based on the individual’s emotional context—further underscoring the dynamic interplay between frontal approach motivation and borderline symptomatology when faced with social exclusion.

Several lines of research beyond the reviewed studies bolster the link between BPD’s clinical features—particularly emotional and interpersonal dysregulation—and altered approach/withdrawal (left vs. right frontal) motivational systems. For instance, the BIS/BAS framework initially proposed by Gray [65] and further operationalized by Carver and White [66] has been influential in explaining how individuals with BPD might exhibit a heightened approach (Behavioral Activation System, BAS) under certain conditions (e.g., perceived abandonment) and abrupt withdrawal (Behavioral Inhibition System, BIS) under others (e.g., shame or fear of closeness). Although most EEG alpha asymmetry work has historically focused on depression or trait anger [66], we know that similar left- vs. right-frontal differences have been implicated in BPD, albeit with less consistency than in depressive disorders [67].

### 4.2. Heightened Arousal and Hypervigilance

Evidence from multiple studies points to a pattern of dysregulated arousal in BPD, typically manifesting as either persistently high vigilance or erratic fluctuations between hyper- and hypo-arousal states. Several investigations, notably [40,44,51], have found reduced alpha-band activity in BPD, particularly under resting or eyes-closed conditions. Because alpha power is often interpreted as an index of cortical “idling” or relaxation, lower alpha suggests a heightened arousal state or “on-alert” status, even when individuals are ostensibly at rest. For instance, in the study [40], BPD participants displayed significantly lower alpha power compared to healthy controls, consistent with elevated emotional reactivity linked to past trauma. At the same time, increased slow-wave activity (delta and theta) appears paradoxical: although in healthy populations, delta/theta elevations typically reflect drowsiness or reduced alertness, in BPD, these patterns often co-occur with signs of heightened distress rather than actual sedation. Studies [44,51], for example, found increased delta power in posterior and frontotemporal regions, positing that these apparently “sleep-like” rhythms may reflect emotional dysregulation or hypervigilance. Similarly, a study [57] observed diffuse theta activity in almost half of all EEGs from BPD patients, supporting the view that these slow waves represent atypical arousal rather than straightforward drowsiness.

EEG-based vigilance staging offers additional insight into how arousal is maintained and regulated. In a study [48], BPD patients stayed in higher vigilance stages (0, A1, A2) significantly longer than healthy controls, underscoring persistent hyperarousal. Conversely, a study [52] showed that while BPD participants began with relatively high vigilance, they showed a steeper decline to lower arousal states (B) than either obsessive–compulsive disorder patients or controls, indicating erratic or poorly regulated arousal dynamics. Notably, in [48], BPD patients also reported feeling more subjectively sleepy despite objectively remaining at higher vigilance levels, suggesting a mismatch between perceived and physiological arousal.

Under stress or pain-inducing conditions, findings continue to support hyperarousal patterns. In a study [45], BPD patients who reported no pain during self-injury (the BPD-NP subgroup) exhibited marked increases in theta activity during a cold pressor test. This increase in theta was associated with dissociation and pain insensitivity, suggesting not so much hypo-arousal as emotional shutdown or dissociative detachment. Together, these results indicate that BPD is characterized by fundamental difficulties in arousal modulation—whether observed as chronically elevated cortical activation, paradoxical slow-wave increases during states of distress, or a mismatch between subjective and objective markers of hypervigilance. Clinically, such dysregulation aligns with emotional lability, heightened stress reactivity, and persistent subjective tension, supporting the notion that hypervigilance is a core neurophysiological component of borderline psychopathology.

The consistent finding of heightened arousal and hypervigilance in BPD is supported not only by the EEG-based studies reviewed here but also by a broader body of psychophysiological and neuroimaging literature. For instance, ambulatory assessment of psychophysiological signals (e.g., electrodermal activity, heart rate) in daily life has shown that individuals with BPD often experience prolonged elevated arousal, with fewer periods of resting-level autonomic activity [68]. From a functional MRI perspective, excessive reactivity in limbic regions (e.g., amygdala, insula) also correlates with the subjective experience of being “constantly on alert” [69,70,71]. This mirrors EEG findings that indicate a mismatch between subjective exhaustion or “sleepiness” and persistently high levels of cortical excitation in BPD (e.g., studies [48,52]).

Moreover, dissociative mechanisms may help explain why delta and theta elevations often coexist with hyperarousal in BPD. Rather than reflecting straightforward drowsiness, these slow-wave increases may partly reflect dissociative “shut-down” phenomena—physiological “escape” routes that co-occur with an inability to deactivate from high arousal [72]. For example, the BPD subgroup that reported pain insensitivity (study [45]) combined elevated theta activity with lower subjective pain ratings, suggesting a state of altered consciousness or detachment rather than genuinely low arousal. These paradoxical co-occurrences (e.g., high alertness in some measures, increased slow-wave activity in others) fit into a model of erratic or disorganized arousal regulation in BPD. The same individual may shift rapidly from hypervigilance to inward-focused, dissociative states without the efficient regulatory processes that characterize healthy individuals.

Clinically, these EEG and psychophysiological data reinforce the notion that hyperarousal and difficulties in downregulating the stress response are central to BPD psychopathology [73]. Interventions like Dialectical Behavior Therapy (DBT) or other skills-based approaches often target these arousal-modulation skills—helping patients detect the early signs of hypervigilance and learn to transition to more adaptive states rather than dissociating or escalating [73]. Future EEG studies could enhance our understanding by systematically pairing real-time EEG measures of arousal (including delta/theta and alpha activity) with momentary self-reports to identify how subjective states, dissociative processes, and objective hyperarousal markers align or diverge across daily life in BPD.

### 4.3. Neural Correlates of Emotion Regulation and Dissociation

A central feature of BPD is profound emotional dysregulation that frequently co-occurs with dissociative symptoms, and several studies point to distinct oscillatory and connectivity markers underlying these phenomena. Theta-band activity emerges as particularly salient. In a study [45], for instance, self-injurious BPD patients who report feeling no pain during self-harm (BPD-NP subgroup) showed marked increases in theta power during a cold pressor test, correlating strongly with elevated dissociation (Dissociative Experiences Scale scores) and reduced pain sensitivity. While heightened theta power often accompanies drowsy or meditative states in healthy individuals, in BPD, it appears to reflect disconnection or emotional shutdown rather than relaxation. Similarly, a study [41] found that baseline FEA correlated with both childhood trauma and dissociative conversion symptoms, suggesting that dysregulation in frontal circuits responsible for approach/withdrawal orientations also links to a propensity for dissociative experiences. These correlations persisted over and above the transient mood changes induced during the experiment, highlighting trait-like neural underpinnings of BPD-related dissociation.

Emotion regulation challenges are further illuminated by studies employing cognitive or feedback-based tasks. In a study [60], BPD participants displayed diminished frontal theta increases and weaker connectivity in the right dorsolateral prefrontal cortex (dlPFC) during a cognitive reappraisal task, implicating frontal theta oscillations in their reduced ability to modulate negative emotions. A similar pattern emerged in the study [59], where simultaneous EEG-fMRI revealed that controls showed stronger theta-band and ACC/dlPFC engagement following loss feedback. In contrast, BPD patients mounted a blunted response to losses and appeared more reactive to gains. A study using microstate analyses [44] also provides complementary evidence: BPD participants showed altered microstate C (linked to default-mode network/self-referential processes) and microstate E (salience/emotion-related network), underscoring how heightened vigilance and impaired self-focused processing may compromise emotional stability. Across these tasks, BPD patients frequently exhibited abnormal reward/loss reactivity (e.g., [49,53])—patterns that can derail adaptive emotion regulation and increase susceptibility to impulsive or self-harming behaviors. Collectively, these results suggest that both chronic dysregulation (as reflected by baseline frontal oscillations linked to childhood trauma and dissociation) and acute task-related deficits (e.g., reduced theta recruitment or atypical feedback responses) play critical roles in the pervasive emotional instability characteristic of BPD.

In line with the findings reviewed, neuroimaging and psychophysiological studies consistently show that emotion regulation and dissociation in BPD involve disrupted frontal-limbic circuitry and aberrant oscillatory dynamics (e.g., theta-band). For instance, fMRI investigations have documented reduced top-down control from the prefrontal cortex over hyperactive limbic regions (amygdala, insula) during negative emotion processing [71,74], complementing the EEG findings of blunted frontal theta recruitment and altered frontal–ACC/dlPFC connectivity (e.g., study [59,60]).

The observation that theta increases co-occur with dissociation and reduced pain sensitivity (as in the study [45]) aligns with clinical models suggesting that BPD patients can alternate between states of hyperarousal and emotional shutdown [72]. These dissociative “shutdown” states may share some oscillatory features with drowsiness or meditation (i.e., elevated theta). Still, they reflect a defensive detachment (or “peritraumatic dissociation” in acute stress contexts) rather than genuine relaxation. This contrasts with healthy individuals, whose theta elevations in restful or meditative states do not typically co-occur with numbing or pain insensitivity.

The cognitive reappraisal deficits (study [60]) and blunted loss feedback response (study [59]) also fit into a larger body of BPD research showing atypical activation in the dlPFC and anterior cingulate cortex (ACC) during emotion regulation [70,74]. Notably, altered microstates (study [44]) suggest a potentially destabilized default-mode network (DMN) and salience network, which may further underlie the self-referential distortions (Microstate C) and hyper-reactivity to emotional cues (Microstate E) seen in BPD. These EEG-based findings converge with fMRI data showing reduced functional connectivity between frontal control regions and limbic structures when BPD patients attempt to regulate negative affect [75].

### 4.4. EEG Abnormalities and Comorbidities

A recurring theme in both older and more recent research is that individuals with BPD tend to show a higher prevalence of nonspecific EEG abnormalities, which often include slow-wave activity, temporal sharp waves, or marginal dysrhythmias. However, the precise clinical implications of these findings remain uncertain, especially given the high rate of comorbid psychiatric disorders in BPD and the overlapping EEG patterns that also appear in other conditions. Studies comparing BPD with dysthymic disorder (e.g., [43]) demonstrate that BPD patients frequently display more frequent and more severe EEG abnormalities, mainly in the form of slow-wave activity localized to temporal or frontotemporal regions. Yet, the correlation between these EEG findings and the clinical severity of BPD remains weak. Similarly, research contrasting BPD with other Axis II disorders (e.g., [58]) indicates that while mild to moderate dysrhythmias may occur somewhat more often in BPD, these differences usually fail to reach statistical significance, thereby complicating any attempt to link such abnormalities exclusively to borderline psychopathology.

Comparisons between BPD and unipolar depression [39,55] also reveal partially overlapping EEG profiles, with both groups commonly showing diffuse slow waves, but BPD sometimes exhibiting distinct posterior sharp waves or spikes. The relationship between EEG abnormalities and the severity of BPD-specific symptoms is equally heterogeneous: while some studies [50,55] report that pronounced abnormalities can align with increased clinical severity (such as fulfilling more DSM-5 criteria or displaying more substantial dissociative and impulsive features), others [43,58] find no consistent associations. Examination of BPD alongside bipolar disorder [46,54] adds another layer of complexity, as conventional EEG metrics can appear similarly abnormal in both conditions. Yet, machine learning and more advanced spectral analyses can sometimes detect subtle differences that yield moderate to high classification accuracy.

Finally, even when epileptiform-like discharges occur in BPD, they seldom meet the criteria for true epilepsy and are more often interpreted as non-specific signs of cortical irritability. The longitudinal data in [57] similarly indicate that some of these abnormalities persist over time without shifting in tandem with clinical states, suggesting a possible trait-like vulnerability. So, EEG anomalies may function as trait-like markers of chronic dysregulation or “cortical irritability.” However, the presence of comorbidities (and the associated fluctuations in mood/anxiety) complicates attempts to parse trait vs. state effects—especially if the co-occurring disorder is episodic (e.g., major depression) or cyclical (e.g., bipolar disorder).

Although many studies have demonstrated a higher prevalence of nonspecific EEG abnormalities in BPD, the interpretation of these findings is frequently complicated by high rates of comorbidity—including mood disorders (e.g., unipolar depression, bipolar disorder), trauma-related disorders (e.g., PTSD), and other personality disorders. Indeed, BPD is sometimes termed a “complex” or “high comorbidity” condition [76], which raises the question: to what extent do EEG markers reflect “pure” BPD pathology versus overlapping features of depression, anxiety, or other conditions?

A practical issue is that many BPD patients receive polypharmacy—commonly, antidepressants, mood stabilizers, or atypical antipsychotics [77]. Even in studies that attempt to control for medication use (e.g., by excluding benzodiazepines or major Axis I disorders), residual confounds often remain. Certain drugs can alter EEG patterns, potentially amplifying (e.g., lithium and anticonvulsants) or attenuating (e.g., antipsychotics) slow-wave activity. As a result, attributing EEG abnormalities solely to BPD becomes challenging if medication effects are not carefully disentangled.

From a nosological perspective, BPD shares emotion dysregulation, impulsivity, and interpersonal instability with certain mood disorders (like bipolar disorder, e.g., studies [46,54]). Subthreshold bipolar features can exist in some BPD presentations, making it hard to attribute abnormal EEG activity uniquely to one diagnosis over the other. Similarly, depression and BPD can both present with diffuse slow-wave changes (as in studies [39,55]), so the presence or severity of depressive episodes could overshadow borderline-specific EEG signatures.

In addition to mood disorders, ADHD is another common comorbidity in BPD [78]. ADHD is associated with EEG’s theta–beta ratio differences [79]. This raises the possibility that increased theta activity occasionally reported in BPD might reflect, at least in part, underlying ADHD symptoms (e.g., impulsivity, inattention) rather than purely borderline-specific neurophysiology. Overlooking these overlapping conditions may inflate EEG findings that seem “unique” to BPD when, in fact, they reflect multifactorial (and often comorbid) processes.

Ultimately, while nonspecific EEG abnormalities may appear more frequently in BPD, they lack the specificity required for diagnostic or prognostic purposes—particularly in the face of substantial comorbidity. Clinicians and researchers thus must interpret EEG findings in conjunction with comprehensive diagnostic and psychosocial evaluations. Future research might employ advanced methods (e.g., source localization, coherence and connectivity analyses, or machine learning classification) to explore further whether there are subtle neurophysiological markers that can differentiate BPD from overlapping disorders when comorbidities and medication status are carefully controlled.

### 4.5. Role of Childhood Trauma and Mentalization Deficits

Multiple studies underscore the critical impact of early adverse experiences on brain function and emotional-cognitive processes in BPD, particularly regarding mentalization capacities—the ability to recognize and interpret one’s own and others’ mental states. Study [40] provides direct evidence that childhood trauma, including emotional abuse, neglect, and bullying, is significantly more prevalent among BPD participants than among healthy controls, correlating with pervasive difficulties in emotional awareness and psychic equivalence (the tendency to conflate one’s internal state with external reality). Altered resting state EEG patterns accompanied these mentalization deficits; for instance, BPD participants showed reduced alpha power in eyes-closed conditions, indicating heightened arousal. Theta power in temporal and parietal regions was positively correlated with impaired emotional awareness. In contrast, delta power in eyes-open recordings was linked to difficulties in emotional expression, implying that adverse childhood experiences might disrupt the neural networks involved in emotional self-reflection and regulation. Research on mentalization [80,81] provides a compelling framework for interpreting the EEG findings of heightened arousal and impaired self-other distinction (e.g., “psychic equivalence” in the study [40]). Mentalization refers to the capacity to reflect upon and understand one’s and others’ mental states. When childhood trauma disrupts the normal development of attachment and self-regulation, this mentalization capacity is compromised, potentially manifesting as the theta-, delta-, and alpha-band EEG alterations linked to hypervigilance and emotion dysregulation [69,72].

The study [41] further demonstrates how early trauma shapes frontal FEA patterns linked to BPD, showing that individuals with high childhood trauma scores exhibit a more substantial rightward shift in frontal alpha power. Although no global differences emerged between BPD participants and controls in baseline FEA, trauma exposure and dissociative symptoms (especially conversion-like phenomena) proved robust predictors of each participant’s asymmetry profile. Moreover, these baseline correlates persisted despite mood induction, suggesting that childhood adversity and dissociative tendencies contribute to stable, trait-like brain-function alterations in BPD rather than transient, state-level shifts.

Studies [40,41] indicate that early abuse, neglect, or bullying co-occurs with pervasive EEG changes—for instance, reduced alpha power (implying chronic arousal) or rightward frontal alpha asymmetry (aligned with withdrawal, dissociation, or anger tendencies). Such patterns echo broader literature linking adverse childhood experiences (ACEs) to persistent changes in stress-response systems [82] and altered frontolimbic connectivity in adulthood [83]. The predictive role of trauma and conversion-type dissociation (study [41]) further underscores how long-standing neural adaptations to early adversity may stabilize into trait-like EEG profiles—especially in networks subserving emotional regulation and self-other differentiation.

Taken together, these findings indicate that childhood trauma and compromised mentalization capacities play a significant role in shaping BPD’s neurophysiological landscape. Early adverse experiences can engender chronic hyperarousal (as revealed by reduced alpha power) and disrupt integrative brain processes (reflected in aberrant theta, delta, and gamma activity), compromising individuals’ ability to distinguish internal states from external reality. Consequently, deficits in mentalization—amplified by childhood trauma—may become entrenched in BPD through maladaptive patterns of cortical activation, contributing to dissociative episodes, unstable self-image, and difficulties in interpersonal relationships.

### 4.6. Resting State EEG Patterns

Taken as a whole, the resting state EEG findings in BPD point to a heterogeneity of neurophysiological profiles while also indicating some convergent themes of heightened arousal, disrupted neural connectivity, and links to emotion dysregulation. Many studies identify a tendency toward increased slow-wave activity (theta and delta), reduced alpha power, or heightened vigilance states, suggesting that BPD involves a persistent hyperarousal rather than a consistently organized EEG pattern.

One prominent thread of evidence is the elevated incidence of slow-wave (theta and delta) activity in BPD, documented in qualitative and quantitative EEG assessments. Several studies [43,57] report higher rates of diffuse slowing among BPD patients relative to controls or comparison groups (e.g., dysthymic disorder). However, the severity of these abnormalities does not always correlate with clinical symptomatology. In line with this, heightened delta and theta power in specific brain regions has emerged as a possible marker for chronic hyperarousal (study [44]) or, in more extreme cases, dissociation-linked pain insensitivity (study [45]). These findings dovetail with microstate analyses showing shorter durations and lower occurrence of resting state microstates connected to self-referential processing (Microstate C) and increased microstates related to salience and alertness (Microstate E), collectively highlighting a predisposition toward heightened vigilance and emotional reactivity (study [44]).

Another consistent pattern involves reduced alpha power and disruptions in typical alpha dynamics. Although alpha asymmetry (i.e., frontal EEG asymmetry) has been a significant focus in some studies [39,41,42], group-level differences in asymmetry are not always robust at rest. Instead, alpha power deficits and alpha coherence reductions appear to align more precisely with symptoms such as difficulties in mentalization, alexithymia, or emotion regulation [40,42]. For instance, a study [40] found that BPD participants showed lower alpha power under eyes-closed conditions, reflecting an inability to downregulate arousal even in a resting state. Similarly, decreased alpha coherence in frontal and temporal regions (study [51]) suggests a breakdown in the coordinated activity that often underlies integrative cognitive and affective processing.

Regarding vigilance regulation, BPD patients have more difficulty achieving lower-arousal EEG stages during extended recordings. Studies [48,52], for example, demonstrate that BPD participants spend more time in higher vigilance states, with reduced flexibility in shifting to restful EEG patterns. Interestingly, these objective EEG measures of hypervigilance can diverge from self-reported states; BPD patients sometimes perceive themselves as tired or sleepy despite electrophysiological indicators of persistent arousal (study [48]). Such findings support the clinical observation that BPD is characterized by chronic tension, hyperalertness, and difficulty “switching off” emotional and physiological reactivity.

It remains challenging to identify a single resting-state EEG marker that universally characterizes BPD. Some studies report higher rates of “definite” EEG abnormalities (e.g., paroxysmal spike-wave patterns, focal slowing) (studies [43,50]), whereas others find only non-specific anomalies or no apparent group differences [47,58]. Methodological factors—such as sample size, diagnostic heterogeneity, medication status, and comorbid conditions—likely contribute to these discrepancies. In addition, BPD symptoms lie on a spectrum of severity: those with multiple borderline criteria or with marked dissociative symptoms appear more prone to EEG anomalies (studies [41,50,55]). Moreover, some investigations suggest that age may interact with BPD symptomatology to amplify EEG abnormalities over time (study [43]), though this requires further longitudinal research.

## 5. Limitations and Future Directions

Despite the growing body of research on EEG correlates of Borderline Personality Disorder, several limitations constrain our current understanding. Likewise, future directions could clarify unresolved issues and enhance the clinical relevance of these findings. Below is a concise overview of key methodological, conceptual, and practical challenges, followed by suggestions for advancing the field.

### 5.1. Methodological Heterogeneity and Small Sample Sizes

A notable challenge in synthesizing the EEG findings across the reviewed studies is the marked variability in the design and execution of research protocols. First, the EEG paradigms differ substantially: some projects record EEG exclusively at rest (often with eyes closed, eyes open, or both), whereas others focus on task-based conditions involving social rejection, cognitive reappraisal, or feedback processing. This diversity in task demands complicates direct comparisons, as resting-state EEG may tap baseline arousal or trait-level patterns. In contrast, task EEG can emphasize state-dependent processes such as emotion regulation or social cognition. Consequently, meta-analytic efforts are hindered by inconsistencies in data-collection procedures, stimulus types, and analysis pipelines (e.g., different frequency-band definitions for alpha, theta, and delta).

A second source of heterogeneity pertains to the EEG metrics themselves. While some investigators rely on qualitative classifications (e.g., standard vs. abnormal EEGs), others use quantitative measures such as spectral power, functional connectivity, microstate analysis, or frontal alpha asymmetry indices. While reflecting innovation, this methodological spread also restricts comparability since each analytic choice can yield different conclusions about underlying neurophysiology. For instance, a study centering on microstate durations might reveal dynamic patterns related to self-referential thought, whereas another that strictly examines frontal alpha asymmetry could focus on approach/withdrawal motivations. Each approach offers distinct insights, yet the lack of standardized protocols can mask or inflate specific findings.

Moreover, a standard limitation is the relatively small sample sizes in many EEG investigations of BPD. Studies often include fewer than 30 BPD participants—sometimes as few as 15—which substantially limits statistical power and complicates the detection of subtle neurophysiological effects. Smaller samples also predispose results to Type II errors (i.e., failing to detect fundamental group differences) and limit the ability to account for subgroup variability (e.g., individuals with high dissociation vs. primarily impulsive presentations). Because BPD is heterogeneous and often involves substantial comorbidity, small-N designs may overrepresent particular phenotypes and unduly skew the observed EEG patterns. A further constraint is that multiple studies rely on patient self-selection or convenience samples from inpatient or outpatient units, raising questions about selection biases. For example, BPD participants who enroll in EEG research might differ in motivation, medication status, or severity of symptoms compared to non-participants.

These methodological discrepancies and small sample sizes underscore the need for larger-scale, multi-site collaborations with unified protocols that foster robust, replicable EEG findings. Recruiting sufficiently powered cohorts, implementing standardized EEG tasks and analysis workflows, and performing sophisticated subgroup analyses could significantly advance our understanding of the distinct neural markers that underlie BPD.

### 5.2. Comorbidity and Medication Confounds

A critical limitation across much of the reviewed EEG research on BPD lies in the substantial rate of comorbidity commonly observed in clinical samples, combined with the widespread use of psychoactive medications. These factors complicate the interpretation of electrophysiological findings, making it difficult to determine whether observed EEG patterns arise from “core” BPD features or instead reflect influences of co-occurring conditions and pharmacological interventions.

First, the issue of high comorbidity stems from the fact that BPD frequently co-occurs with other psychiatric diagnoses, notably major depressive disorder (MDD) [84], bipolar disorder (BD) [85], anxiety disorders [86], PTSD [87], and substance use disorders [88]. Because each of these conditions can exhibit overlapping EEG signatures—such as elevated slow-wave power or diminished alpha coherence—EEG “abnormalities” initially attributed to BPD may reflect underlying depression, subthreshold bipolar traits, or anxiety-driven hyperarousal. This confounds attempts to isolate a neurophysiological “essence” of borderline pathology. In many instances, diagnostic protocols may fail to distinguish subgroups of BPD patients with markedly different comorbidities. These obscuring patterns might be specific to impulsivity, dissociation, or other BPD-relevant dimensions.

Second, medication use is widespread among individuals with BPD. Polydrug regimens often include antidepressants, mood stabilizers, antipsychotics, or benzodiazepines, each of which can alter baseline EEG rhythms. For example, mood stabilizers such as lithium or valproate can amplify slow-wave activity (delta and theta) and fast activity (beta) [89], while antipsychotics sometimes blunt overall cortical reactivity [90]. Consequently, any reported EEG phenomena might reflect drug-induced changes rather than borderline-specific neurophysiology. Furthermore, even studies that attempt to recruit unmedicated BPD participants rarely control for prior medication exposure; residual or carry-over effects may persist if discontinuation intervals are brief. Selection biases may also emerge if only those with less severe pathology can safely discontinue pharmacotherapy, further skewing the sample.

Together, comorbidity and medication confound the specificity and generalizability of findings. On the one hand, comorbid disorders can mask or mimic borderline EEG markers; on the other, pharmacological treatments can create alterations that may look like “core” BPD patterns. Interpreting which portion of the variance truly represents borderline psychopathology becomes highly challenging, especially as most studies either exclude specific comorbidities arbitrarily or fail to assess them systematically. A similar limitation arises regarding medication regimens, where varied drug histories or ongoing prescriptions impede clean inferences about the underlying neural substrate. As a result, the field lacks a firm consensus on which EEG anomalies are genuinely tied to BPD-related dysregulation vs. which stem from overlapping conditions or pharmaceutically induced shifts.

Overcoming these confounds requires more careful screening and design. Future research would benefit from larger, well-characterized samples in which comorbidities are thoroughly documented and modeled in statistical analyses, allowing for more nuanced identification of BPD-specific EEG patterns. Where ethically feasible, extending medication washout periods or systematically comparing medicated and unmedicated groups could illuminate the unique effects of pharmacological interventions. Multimodal approaches—incorporating structured interviews, additional neuroimaging modalities, and psychophysiological measurements—could further help disentangle the signatures of BPD from those of commonly co-occurring disorders. Ultimately, addressing these confounds should clarify whether the EEG patterns frequently linked to BPD are indeed distinct indicators of borderline psychopathology or whether they reflect more general neurophysiological disruptions associated with complex psychiatric presentations.

### 5.3. Task-Specific vs. Resting State Discrepancies

A further limitation in the literature on EEG correlates of BPD involves inconsistent findings when comparing resting state paradigms to task-based procedures. While some studies report apparent group-level differences in resting EEG—for instance, diffuse slow-wave increases or reduced alpha activity—others find no distinctive resting-state abnormalities. Instead, certain patterns only emerge under specific tasks, such as social rejection, pain induction, or cognitive reappraisal. This discrepancy complicates the interpretation of BPD-related EEG results in two main ways.

First, the reliance on brief, often single-condition assessments of EEG activity may overlook the dynamic nature of borderline psychopathology. BPD is characterized by fluctuating mood states, rapid changes in arousal levels, and marked sensitivity to interpersonal triggers. In many resting state protocols, participants sit quietly with eyes closed or open, which may not activate the same neural processes underlying the core emotional and interpersonal disruptions typical of BPD. Consequently, a participant might appear within normal limits on resting measures yet exhibit significant electrophysiological deviations under a task that elicits fear of rejection, frustration, or other stressors. Conversely, those who show aberrant baseline rhythms might display relatively modest further shifts when confronted with experimental tasks, making it difficult to predict how those individuals respond to real-world triggers.

Second, the tasks themselves vary widely in nature and complexity. Some use standardized cognitive paradigms (e.g., gambling tasks and basic emotion regulation instructions). In contrast, others expose participants to more ecologically relevant stimuli, such as social exclusion (Cyberball) or painful sensations (cold pressure tests). These methodological differences can create substantial variability in the type of EEG anomalies observed. For instance, alpha asymmetry linked to approach or withdrawal tendencies may be most apparent under interpersonal threat or rejection conditions, whereas variations in theta or delta bands could be more easily detected during a more straightforward resting state condition. Moreover, subtle differences in task instructions or timing parameters may explain why one study uncovers significant group differences while another does not. This task-versus-resting distinction raises the question of how best to capture the clinically pertinent aspects of BPD using EEG. Some forms of borderline pathology—particularly those involving impulsive aggression or self-injurious behaviors—may be better evoked in response to specific stress tasks or emotionally salient stimuli.

In contrast, if sufficiently long and well-structured, chronic hyperarousal or background dysregulation might be more detectable during a passive, eyes-closed baseline. The fact that specific individuals with BPD report subjective hypervigilance yet show inconclusive findings at rest suggests that many neurophysiological disruptions may be state-dependent or context-contingent rather than purely trait-like. Conversely, one must also recognize that repeated or extended task sessions could result in fatigue or habituation, obscuring the phenomena under scrutiny.

A further consideration is that the type of tasks used—particularly those mimicking social challenges—can have high ecological validity for BPD symptoms yet introduce additional confounds, such as frustration effects or differences in task engagement. Although real-life triggers can powerfully elicit borderline coping styles, participants might respond inconsistently, depending on individual personality features, momentary mood, or prior interpersonal experiences. Studies employing standardized tasks often assume a uniform emotional response, whereas BPD presentations are highly heterogeneous in practice. The tension between task-based specificity and resting-state simplicity thus complicates a unified approach to EEG research in borderline populations.

In light of these issues, future research could benefit from combining carefully designed resting state measures with ecologically valid experimental conditions, allowing investigators to observe how baseline dysregulation interacts with acute stress or emotion regulation challenges. Additionally, multi-session protocols incorporating real-time self-report (e.g., momentary affect ratings) or ambulatory EEG could better capture the fluctuating nature of borderline symptoms across diverse contexts. By integrating resting state and task-induced measures within the same participants, researchers might clarify how stable trait-level EEG patterns compare to dynamic, state-dependent shifts in neural activity. This dual approach would also help distinguish which aspects of BPD psychopathology are continuously active—detectable even at rest—and which are predominantly triggered by salient external stimuli.

### 5.4. Underexplored Mechanisms: Childhood Trauma, Mentalization, and Dissociation

Another significant gap in the existing EEG literature on BPD concerns the underexplored roles of childhood trauma, impaired mentalization, and dissociative processes—factors that often appear central to borderline psychopathology. Although multiple studies highlight how adverse developmental experiences can shape chronic hyperarousal and emotion dysregulation, much remains unclear about the specific neural mechanisms through which early trauma and mentalization deficits become embedded in adult EEG patterns, particularly regarding dissociation-related phenomena.

A first challenge arises from the high prevalence of trauma in BPD populations: emotional abuse, neglect, bullying, or more severe experiences can disrupt standard attachment and self-regulatory capacities [2,91,92]. Indeed, several investigations (e.g., studies [40,41]) link these adverse experiences to alterations in alpha activity (suggesting persistent hypervigilance) and shifts in frontal asymmetry (e.g., a rightward bias correlating with withdrawal or dissociative tendencies). Nonetheless, few studies systematically dissect the type, severity, or developmental timing of trauma about specific EEG abnormalities—such as distinct slow-wave patterns or cortical irritability. As a result, important questions persist. For instance, do experiences of childhood emotional neglect predict a different electrophysiological signature than physical abuse? Does trauma occurring at earlier developmental stages yield more entrenched EEG changes than later trauma exposures? Do cumulative adverse experiences result in greater cortical instability?

Second, research on mentalization deficits in BPD remains relatively new in the EEG domain. Mentalization refers to the capacity to interpret one’s own and others’ behaviors in terms of underlying mental states [93]; when severely compromised, as commonly found in BPD [94], individuals may confuse inner emotional experiences with external reality (“psychic equivalence”) or struggle to reflect on their motivations [95]. While several studies (e.g., [40]) report correlations between mentalization impairments and specific EEG markers (like elevated theta or reduced alpha), the detailed neural pathways underlying these links remain ambiguous. It is also unclear whether improving mentalization skills (e.g., through Mentalization-Based Treatment) might normalize certain EEG features, such as frontotemporal coherence or alpha power under resting conditions, thereby clarifying the causal direction of the mentalization–EEG relationship.

Third, dissociation stands out as a recurring yet incompletely explained phenomenon in BPD. In some cases, dissociative symptoms manifest as “conversion-type” experiences (e.g., functional neurological disruptions), while in others, they involve profound detachment or pain insensitivity [96]. Studies like [41,45] show that elevated theta, linked to both hyperarousal and a “shut-down” state, correlates strongly with dissociative indicators and reduced pain perception. This suggests a possible interplay between intense arousal circuits and dissociative “escape” mechanisms. However, additional research is needed to specify how these dissociative EEG signatures differ from those of other disorders involving dissociation (e.g., complex PTSD) or whether specific BPD subgroups (e.g., those with chronic depersonalization) display more pronounced theta/delta abnormalities. Furthermore, whether these electrophysiological patterns are consistently observed in daily life or are primarily triggered by acute stress or interpersonal conflict remains an open question.

A final complication is that childhood trauma, mentalization deficits, and dissociation are often interwoven [97,98]. For example, the same individual might develop dissociative coping strategies in response to severe trauma, which in turn impedes mentalization and fosters chronic hypervigilance—each factor potentially leaving its own EEG “footprint.” Teasing apart these layered influences would require well-powered, multivariate designs that measure trauma histories in detail, evaluate mentalization capacities using robust clinical instruments, and track dissociative symptoms using standardized scales. Researchers could then relate these dimensions to EEG parameters—both at rest and under tasks that challenge or stimulate mentalization abilities—while controlling for co-occurring disorders and medication effects. Such an approach might reveal discrete sub-pathways: for instance, individuals with extreme dissociation might show a unique slow-wave profile that diverges from those with severe emotion dysregulation but minimal dissociation.

In summary, although early trauma, mentalization deficits, and dissociation shape the clinical trajectory of BPD, their precise neurophysiological correlates remain insufficiently understood. Unraveling these mechanisms will likely require integrative, longitudinal studies that follow at-risk individuals over time, mapping the emergence of borderline features alongside evolving EEG changes. Coupled with treatment studies that assess whether improvements in mentalization or reductions in dissociation lead to corresponding shifts in cortical activity, such research could clarify how deeply ingrained adversities and self-other processing deficits become codified in the adult BPD brain. By addressing these questions directly, future investigations stand to refine both the theoretical models of borderline pathology and the potential for EEG-informed interventions.

### 5.5. Toward Personalized EEG Biomarkers and Treatment

A final yet pivotal direction in EEG research on BPD lies in developing individualized biomarkers that could inform more tailored clinical interventions. While numerous studies point to elevated slow-wave activity, altered alpha power, or shifts in frontal asymmetry, these patterns often vary substantially at the individual level and overlap considerably across different patient subsets. This heterogeneity suggests that a “one-size-fits-all” EEG signature for BPD is unlikely and that precision psychiatry approaches—integrating advanced data analytics with nuanced clinical phenotyping—may prove more fruitful.

One emerging strategy involves machine learning techniques that detect subtle, multidimensional EEG features that might elude standard group-mean analyses. Instead of relying on broad spectral band differences, machine learning classifiers (e.g., k-Nearest Neighbors [99], Support Vector Machines [100], or decision trees [101]) can incorporate multiple frequency bands, connectivity measures, and temporal dynamics to classify individuals or predict outcomes. As demonstrated in [54], the ability of wavelet-based EEG features to differentiate BPD from Bipolar Disorder type II underscores the potential power of these computational tools. By scaling up sample sizes and enriching the input data with clinical variables (e.g., impulsivity scores, dissociation measures, trauma histories), researchers may identify EEG-derived subtypes of BPD, each with distinctive patterns of neural dysregulation. Such subtyping could directly inform treatment personalization. For instance, patients showing pronounced dissociative theta surges (as observed in [45]) might benefit most from interventions targeting dissociation reduction (e.g., grounding techniques, sensorimotor approaches). At the same time, those with marked frontal alpha asymmetry shifts in response to perceived rejection could require specialized social cognition or rejection-sensitivity protocols. Similarly, identifying a BPD subgroup that exhibits frequent paroxysmal or sharp-wave activity might prompt closer evaluation for subthreshold seizure tendencies or vulnerability to stress-induced episodes, resulting in different medication strategies. Although these applications remain largely theoretical, they illustrate how individualized EEG signatures might eventually guide a more granular approach to borderline interventions.

A second personalization aspect involves tracking EEG changes over therapy to monitor real-time shifts in arousal regulation, limbic reactivity, or frontal-limbic connectivity. If baseline recordings reveal excessive delta/theta in specific cortical regions, clinicians and patients could visualize EEG progress as they implement emotional regulation skills (e.g., in Dialectical Behavior Therapy or Mentalization-Based Treatment). This “neurofeedback-like” paradigm could help reinforce adaptive strategies, providing clinicians and patients with tangible evidence of improved neural regulation. However, establishing robust normative data for borderline populations—and validating EEG improvements against meaningful clinical outcomes (e.g., reduced self-harm or improved interpersonal functioning)—remains an essential prerequisite.

Moreover, longitudinal, multi-time point designs are needed to separate fleeting EEG fluctuations from stable, trait-level dysregulation. By following individuals across different symptomatic periods and treatment phases, researchers could determine if specific neural markers (like persistent alpha suppression) are unresponsive to standard care or if particular subgroups show dramatic EEG normalization once core trauma issues are addressed. Such findings would help refine when, how, and for whom EEG measures can guide therapeutic adjustments or signal emerging relapse risks.

Still, substantial work lies before EEG-based personalization becomes a clinical reality for BPD. Medication confounds, comorbidity complexities, and methodological inconsistencies must be addressed to ensure that identified EEG subtypes or predictive signatures truly capture borderline-specific processes rather than general psychiatric dysfunction. Additionally, interdisciplinary collaborations—combining data science, clinical psychology, and neurobiology—are vital to move beyond proof-of-concept algorithms and into robust, reproducible biomarker pipelines. In parallel, ethical considerations surrounding individual-level neurodata (e.g., privacy and interpretation complexities) will require careful navigation if EEG-informed treatments become more widespread.

Overall, a personalized EEG approach holds promise for enhancing the precision of BPD diagnosis, prognosis, and therapy allocation. Future research can shift away from broad, group-level averages by discovering nuanced neural phenotypes through machine learning, monitoring evolving EEG indices during treatment, and systematically linking these patterns to functional outcomes. This next-generation paradigm would respect the profound heterogeneity of BPD presentations while leveraging cutting-edge electrophysiological insights to optimize interventions, thereby marking a significant step forward in the clinical management of borderline psychopathology.

### 5.6. The Lack of Formulated Definitions of EEG Frequency Bands

A notable methodological challenge across the reviewed studies is the absence of a consistent framework for defining and labeling EEG frequency bands. Although the field broadly recognizes major frequency ranges (delta, theta, alpha, beta, and gamma), the precise cutoffs for each band vary widely from one investigation to another. For example, in some studies, delta is defined as 0.5–4 Hz, whereas others adopt 1–4 Hz or 0.75–3 Hz. Similarly, theta can start as low as 3 Hz or as high as 4 Hz. Its upper boundary may range anywhere between 6 Hz and 8 Hz. Discrepancies in alpha-band definitions are likewise evident: specific authors define alpha as 8–12 Hz, while others extend alpha up to 13 Hz or subdivide it into alpha1 (8–10 Hz) and alpha2 (10–12 Hz). Beta and gamma bands are also split into multiple subranges in some studies—beta1, beta2, beta3; gamma1, gamma2, and high gamma—each with its unique thresholds. Such inconsistencies complicate direct comparisons of findings, particularly when attempting to synthesize results via meta-analyses or systematic reviews. Even slight differences (e.g., a 1–2 Hz shift in alpha or beta boundaries) can alter the interpretation of power changes, coherence measures, or asymmetry patterns. These variations become especially salient when studying clinical populations, such as patients with BPD, who already exhibit heterogeneity in comorbidities, medication status, and symptom presentation. Differences in frequency band definitions add another layer of variability, making it difficult to determine whether observed discrepancies across studies stem from genuine neurophysiological differences or merely reflect divergent analytical conventions.

Additionally, some investigations in this review subdivide “traditional” frequency ranges into narrower bands (e.g., alpha1 vs. alpha2, beta1 vs. beta2 or beta3). While these subdivisions may capture more nuanced information about cortical dynamics, they also introduce new complexities. The boundaries between sub-bands vary by study, and not all researchers agree on how best to parse the frequency spectrum for clinical populations. As a result, one study’s alpha1 band could overlap with another’s alpha2 or even the high-theta range, hampering meaningful cross-study comparisons.

Moving forward, more explicit consensus guidelines for EEG frequency band definitions in clinical research would improve reproducibility and comparability. Although some international guidelines exist, many researchers adapt these standards to specific research questions or equipment parameters, highlighting the need for broader field-wide coordination. Establishing a standard set of definitions—or at least requiring explicit reporting and justification of chosen frequency cutoffs—would allow future BPD studies (and clinical EEG research more generally) to build a more coherent cumulative knowledge base. This effort would also foster more robust meta-analyses, ultimately advancing our understanding of the nuanced neurophysiological patterns underlying BPD and related psychiatric disorders.

### 5.7. Integrating Cross-Cultural and Developmental Perspectives

Despite accumulating evidence on EEG correlates of BPD, most research has been conducted in Western or high-income settings, which limits the generalizability of findings to diverse cultural groups whose social norms, family structures, and stressors may vary markedly. Cultural factors could influence how borderline traits emerge or manifest at the neurophysiological level [102,103], yet relatively few studies have examined BPD across varied sociocultural contexts. In addition, adolescence and early adulthood are critical windows for developing BPD symptoms [104]. Still, many investigations focus on adult samples or combine participants across a broad age range without conducting age-specific analyses. This omission is problematic because neural organization and brain maturation differ substantially between adolescent and adult populations, and EEG markers linked to borderline features could shift over time. Moving forward, cross-cultural investigations with ethnically and geographically diverse cohorts would help clarify whether EEG anomalies such as heightened theta or reduced alpha power are consistently observed or, instead, culturally contingent. Likewise, longitudinal designs tracing individuals from early adolescence into adulthood could reveal how borderline traits and associated EEG signatures develop together—especially in high-risk adolescents who show preliminary signs of impulsivity, affective instability, or dissociation. Adapting paradigms for cultural relevance (for example, tailoring social rejection or emotion-induction tasks) and tracking changes in EEG patterns across critical developmental transitions would strengthen our understanding of when, how, and in whom these neurophysiological disruptions arise, ultimately improving the clinical detection and targeted treatment of BPD in diverse populations.

### 5.8. Broadening Multimodal Approaches and Collaboration

While one BPD study incorporates fMRI alongside EEG, most still rely on electrophysiological measures in isolation, a single-modality focus that can overlook critical structural or functional insights from brain imaging or physiological data such as heart rate variability or cortisol levels. Furthermore, limited collaboration across research disciplines and centers curbs large-scale investigations that could yield more diverse patient samples and robust data-sharing protocols. Moving forward, combining EEG with MRI, PET, or other physiological measures (e.g., skin conductance, heart rate variability) can offer a deeper understanding of how cortical dynamics interface with autonomic arousal and subcortical activity in BPD; for instance, coupling EEG microstate analysis with resting-state fMRI may clarify how altered microstates map onto specific neural networks. Establishing multisite consortia and adhering to open-science frameworks can expand statistical power, promote replication, and standardize protocols for identifying reliable EEG biomarkers of BPD. Additionally, advanced machine learning approaches that integrate EEG signals with other neuroimaging or psychophysiological data could reveal multidimensional biomarker profiles that capture the disorder’s heterogeneity more precisely than single-modality approaches, ultimately guiding more targeted intervention strategies.

### 5.9. EEG in BPD—Chance for Neurofeedback?

We have shown that BPD is associated with changes in resting EEG activity. Can these changes be altered through conscious brainwave training? EEG neurofeedback (NF) is a closed-loop operant-conditioning method: ongoing scalp EEG is acquired, processed in real time to extract a target feature (e.g., band power, band ratios, coherence, or slow cortical potentials), compared to an adaptive threshold, and then converted into visual or auditory rewards. Over repeated trials, reinforcement contingencies shape up- or down-regulation of the target neural signal. Typical pipelines include online artifact mitigation, short-window spectral estimation, threshold “shaping,” and low-latency feedback [105,106].

Unfortunately, to date, the effectiveness of EEG-based neurofeedback in treating BPD has not been studied. This represents a significant gap in the application of this therapeutic intervention, but also a significant opportunity to discover whether this type of neural behavioral training can alleviate BPD symptoms. Here are candidate EEG-neurofeedback protocols that follow directly from the EEG patterns reported in included studies.

(1) Arousal down-shift (alpha/SMR up, slow-waves down)—for chronic hyperarousal

Rationale: The review repeatedly notes reduced alpha and elevated slow-wave (theta/delta) with unstable vigilance in BPD, consistent with “always-on” tension. Protocol: eyes-open training to increase posterior alpha (8–12 Hz; Pz/Oz) or SMR (12–15 Hz; Cz/C3/C4) while inhibiting theta/delta bursts and brief high-beta spikes (muscle). Vigilance-stabilization framing is appropriate given the lower A-states and more A → B transitions seen in BPD.

(2) Frontal midline theta “reappraisal support”—for emotion regulation

Rationale: During cognitive reappraisal, people with BPD showed smaller increases in frontal theta and weaker frontal connectivity; theta level correlated with reappraisal success. Protocol: task-coupled NF at Fz/FCz to up-train 4–7 Hz while participants actively reappraise negative IAPS images (or DBT skills practice), rewarding brief theta increments within a low-artifact window. (A related reward-feedback study also found altered theta during loss feedback, supporting a theta-centric target during control-demanding tasks.)

(3) FAA balancing—for approach/withdrawal and rejection sensitivity

Rationale: Frontal alpha asymmetry (F3/F4) moderated the link between BPD features and rejection sensitivity in adolescents (greater left-frontal = higher sensitivity under high BPD traits), and trauma/dissociation history predicts right ward shifts. Protocol: alpha-band NF at F3/F4 to reduce extreme asymmetry (move toward symmetry or context-appropriate direction), paired with social-cognition/rejection-exposure tasks to make learning state-specific.

(4) Reward-signal normalization (beta/delta)—for feedback sensitivity

Rationale: In adults, low-beta (≈15 Hz) gain > loss responses tracked BPD symptom severity; in adolescent females, reward-related delta was blunted. Protocol: task-based NF during monetary guessing to (a) dampen excessive low-beta bursts to gains (central/parietal sites) or (b) enhance phasic delta to rewards in youth, aiming to reduce biased valuation/reactivity.

(5) Coherence/connectivity strengthening—for disrupted networks

Rationale: Studies reported reduced alpha coherence and altered delta/theta coherence in BPD, consistent with weaker long-range integration. Protocol: alpha-coherence NF (fronto-parietal; Fz–Pz/F3–P3/F4–P4) with artifact-robust estimators; reward sustained coherence plateaus rather than spiky transients.

Because BPD is a severe disorder, future research and potential treatment trials should include a minimum of 20 neurofeedback sessions, each lasting at least 30 min.

## 6. Conclusions

Although the reviewed studies employ diverse EEG methodologies and examine a wide array of clinical presentations, several convergent themes emerge that illuminate the neurophysiological underpinnings of BPD. First, many investigations point to pervasive hyperarousal and difficulties in downregulating heightened emotional or physiological states. These findings frequently manifest as reduced alpha power, increased slow-wave activity (theta/delta), and prolonged or rigid vigilance stages—patterns that underscore the chronic tension and “on-alert” status widely observed in BPD. Second, research on frontal EEG asymmetry highlights the interplay between approach and withdrawal tendencies, showing that individuals with BPD may shift toward left-frontal activity (increased approach/hostility) or right-frontal patterns (dissociative withdrawal) depending on contextual stressors such as social rejection or internal triggers like trauma-related memories.

A third consistent thread is the strong influence of childhood trauma and deficits in mentalization, as well as dissociative processes. Adverse developmental experiences correlate with trait-like changes in EEG—most notably reduced alpha power (reflecting sustained hypervigilance) and increased theta power (associated with dissociation or emotional “shut-down”). These findings suggest that early trauma may exert enduring effects on frontolimbic networks integral to emotion regulation and self-other distinction. Nonetheless, studies vary in how directly and consistently they link these EEG anomalies to specific BPD symptoms, indicating that no single “universal” EEG marker of BPD exists.

A central challenge in this literature is methodological heterogeneity. Sample sizes often remain small; analytic methods, definitions of frequency bands, and experimental protocols vary widely; and medication confounds or comorbid conditions are not always systematically addressed. Moreover, while some work documents resting state abnormalities, others observe EEG deviations only under emotionally charged tasks (e.g., social rejection, pain induction, or cognitive reappraisal). These differences highlight the dynamic nature of BPD, in which trait-level dysregulation may be overshadowed or amplified by state-dependent triggers.

Overall, EEG findings in BPD underscore the disorder’s complex interplay of hypervigilance, emotional dysregulation, dissociation, and, for many, the enduring impact of childhood trauma. From a clinical perspective, EEG holds the potential for identifying subgroups of patients—such as those with pronounced dissociative tendencies or heightened approach-related hostility—who might benefit from targeted interventions. Future work combining standardized EEG paradigms with multimodal assessment (e.g., fMRI, heart rate variability, cortisol) and advanced analytical techniques (e.g., machine learning, source localization) will likely yield more robust and personalized biomarkers. By clarifying which EEG indices correspond to stable traits versus context-driven states, researchers and clinicians can better capture the heterogeneity of BPD and refine interventions that address its core neurobiological mechanisms.

## Figures and Tables

**Table 1:** Summary of the included EEG studies on Borderline Personality Disorder.

Study	Participants	EEG Method/Design	Additional Tasks/Measures	Main EEG-Related Findings
Beeney et al. [39]	- 23 BPD, 13 MDD, 21 HC (all female, right-handed; 18–60 yrs) - BPD median age ≈ 31.84	- 8 min baseline (eyes open/closed) - 2 min post-task resting EEG - Frontal alpha asymmetry (8–13 Hz), 11 frontal electrode pairs	- Cyberball social rejection task - Beck Depression Inventory-II (BDI-II), PANAS-X - Hostility and rejection sensitivity measures	- Baseline: No significant group differences, slight right-frontal asymmetry in MDD, balanced/left in BPD and HC - Post-rejection: Marked left-frontal asymmetry in BPD (approach) vs. right-frontal in MDD (withdrawal) - Leftward asymmetry correlated with higher hostility in BPD
Yun et al. [40]	- 45 BPD (mean age ≈ 26) - 15 HC (age and gender matched)	- 5 min resting state EEG (eyes closed/open) - Power spectral density (PSD) in delta–gamma bands	- PROVE-ACE (childhood trauma) - PROVE-MC (mentalization)	- BPD showed reduced alpha (EC), indicating hyperarousal - Theta (EC) correlated with emotional awareness deficits - Delta (EO) linked to difficulties in emotional expression - Gamma (EO) negatively correlated with psychic equivalence
Popkirov et al. [41]	- 26 BPD (median age 31.42) - 26 HC (age/gender/handedness matched)	- Resting state EEG (eyes open), 32 electrodes - Pre- and post-mood induction - Frontal EEG asymmetry (FEA) = ln(Right α) − ln(Left α)	- Childhood Trauma Questionnaire (CTQ) - Dissociative Experiences Scale (DES), BDI-II - Mood induction with negative/neutral IAPS images	- No overall FEA group difference at baseline - Both groups had a rightward FEA shift post-induction - BPD showed higher trauma/dissociation - Childhood trauma and dissociative symptoms predicted baseline FEA in BPD (51% variance)
Flasbeck et al. [42]	- 37 BPD females (mean age ≈ 26.8) - 39 HC females (age-matched)	- 4 min resting state EEG (eyes closed), 32 electrodes - FEA = ln(Right α) − ln(Left α)	- Toronto Alexithymia Scale (TAS-20) - BDI-II, SCL-90-R	- No overall FEA difference BPD vs. HC - Within BPD, FEA correlated with alexithymia (especially “describing feelings”) - Depression and general psychopathology not linked to FEA
Snyder et al. [43]	- 37 male BPD (DSM-III, stringent criteria) - 31 male dysthymic disorder	- 30 min resting EEG + 30 min sleep recording - Hyperventilation and eye-open/closed cycles	- Neurological evaluations - Abnormalities rated blind by neurologists	- 19% of BPD had marginally abnormal, 19% definitely abnormal EEG - Most frequent abnormality: slow-wave activity (19% in BPD vs. 3% in dysthymia) - No correlation with symptom severity, but older BPD had more severe abnormalities
Deiber et al. [44]	- 16 BPD (15F; mean age ≈ 25) - 16 HC (10F; mean age ≈ 29.6)	- High-density (256-channel) resting state EEG (3 min, eyes closed) - Microstate analysis and spectral power (delta–beta)	- Montgomery-Åsberg Depression Rating Scale (MADRS) - Affective Lability Scale (ALS)	- BPD showed reduced alpha and increased delta in posterior-midline sites - Microstate C (DMN-related) reduced in BPD; Microstate E (salience-related) increased - Microstate changes correlated with affective lability
Russ et al. [45]	- 22 BPD-P (pain during self-injury), 19 BPD-NP (no pain), 15 MDD, 20 HC - All female (BPD-P age ≈ 31, BPD-NP ≈ 26)	- 4 min resting state EEG (eyes closed) + 4 min cold pressor test (CPT) - 16 electrodes, delta–beta power	- Dissociative Experiences Scale (DES) - Pain ratings every 15 s during CPT	- BPD-NP showed lowest pain ratings and highest theta increases - Theta correlated with dissociation and inversely with pain - BPD-P had moderate theta increases; depressed group similar to HC
Arikan et al. [46]	- 25 BPD (mean age ≈ 26.36) - 75 BD (33.56), 11 HC (32.33)	- 7 min resting state EEG (eyes closed) - Quantitative EEG (qEEG), multiple bands (delta–gamma)	- Retrospective chart review	- Significant group differences vs. HC in delta, theta, beta, gamma - No post hoc differences between BPD and BD - Suggests shared electrophysiological patterns in BPD & BD
Ogiso et al. [47]	- 18 BPD (18–30 yrs, mean ≈ 23.1) - 21 non-BPD patients (mean ≈ 23.0)	- Resting EEG with eyes open/closed, hyperventilation, photic stimulation (3 min each)	- Diagnostic Interview for Borderline Patients (DIB) - DSM-III criteria	- No unique EEG abnormalities distinguishing BPD - Across all patients: certain spike/slow-wave patterns correlated with impulsivity and interpersonal issues, but not specific to BPD
Kramer et al. [48]	- 40 unmedicated BPD (26F, 14M; mean age ≈ 27.95) - 42 HC (23F, 19M; mean age ≈ 27.98)	- 20 min resting state EEG (eyes closed) - EEG-vigilance analysis (VIGALL 2.0)	- Structured clinical interviews, borderline symptom measures, impulsivity, depression, childhood trauma, sleep quality	- BPD exhibited consistently higher vigilance stages (less transition to drowsiness/sleep) - Greater subjective sleepiness despite hyperarousal - Reduced lability in EEG-vigilance (rigidly high arousal)
Schauer et al. [49]	- 18 BPD (mean age ≈ 28.28) - 22 HC (26.27)	- 64-channel EEG - Time-frequency analysis (alpha, low-beta) during gain/loss feedback	- Two-choice gambling task (low vs. high stakes) - BPD symptom severity (BSL-23)	- Alpha power: no group difference - Low-beta power: BPD showed increased gain > loss response - Low-beta gain-loss difference correlated with greater BPD symptom severity
Shankar et al. [50]	- 60 BPD (18–40 yrs) - 60 HC (age-matched)	- 40 min EEG with standard activation (hyperventilation, photic stimulation)	- Symptom severity based on DSM-5 BPD criteria	- 31.7% BPD vs. 3.3% HC had EEG abnormalities - Frontal/temporal, right-sided predominance - Greater severity (≥9 criteria) linked to higher epileptiform discharges
Rezaei et al. [51]	- 7 BPD (screened via MCMI-III) - 7 HC (university students, ~22 yrs)	- 5 min resting state EEG (eyes closed), 21 channels - Power spectral density (PSD) and coherence in delta–gamma	- MCMI-III screening - Artifacts processed in EEGLAB (MATLAB)	- BPD had increased delta power (frontotemporal/parietal) - Reduced alpha power (frontal/central) and lower alpha coherence - Suggestive of disrupted cortical connectivity
Hegerl et al. [52]	- 20 BPD (mean age ≈ 27) - 20 OCD, 20 HC (age/gender matched)	- 5 min resting state EEG (eyes closed) - Computer-assisted EEG-vigilance staging (A1–A3, B, etc.)	- Structured interviews, impulsivity and borderline measures	- BPD showed lower vigilance state A vs. OCD and HC - Greater shifts to lower vigilance states (B) and more artifact segments - Vigilance instability remained significant after adjusting for artifacts
Stewart et al. [53]	- 35 BPD females (13–23 yrs, median age ≈ 17.59) - 33 HC (female)	- EEG recorded during guessing task (monetary gain/loss) - ERP and time-frequency analysis (theta and delta)	- Structured Clinical Interview for DSM-IV Axis II - No neurological disorders	- BPD showed significantly lower delta power in response to rewards - No group differences in theta power or loss-related delta power
Nazari et al. [54]	- 25 BPD (median age ≈ 30.64) - 20 BD II	- 10 min EEG (eyes-closed/open), 21 channels - Spectral/wavelet features (delta–beta)	- WCST, Integrated Cognitive Assessment (ICA) - Machine learning (KNN, SVM, decision trees)	- Strong EEG feature differences between BPD and BD II - Best classification (KNN) reached ~89% accuracy - Cognitive tests contributed minimally to differentiation
Cowdry et al. [55]	- 39 BPD (mean age ≈ 20.2) - 20 unipolar depression (UP)	- Clinical EEG (16 electrodes, bipolar and monopolar), hospitalized	- Diagnosed before DSM-III - Research Diagnostic Criteria for major depression (UP)	- BPD had higher rate of EEG abnormalities (46%) vs. UP (10%) - Posterior sharp/spike-wave discharges common in BPD - Overlap with complex partial seizure (CPS) and episodic dyscontrol symptoms
Pop-Jordanova et al. [56]	- 10 BPD (5M/5F, mean age 20.4) - 10 HC (6M/4F, mean age 24.2)	- 5 min resting EEG (eyes open) - Coherence analysis (delta–beta2)	- DSM-5 criteria for BPD	- BPD showed lower delta/theta coherence across regions - No alpha/beta coherence differences - Non-significant increase in delta/theta power in BPD
De la Fuente et al. [57]	- 20 BPD inpatients (14F/6M; mean age 32.4) - Double-blind CBZ vs. placebo (n = 10 each)	- 17-channel EEG at baseline (day 0), day 16, day 32 - Resting + hyperventilation and photic stimulation	- DSM-III-R criteria - 10-day washout, 32-day treatment	- 40% showed diffuse slow activity (theta ± delta) at baseline - No focal or epileptiform discharges - CBZ did not significantly reduce EEG abnormalities
Cornelius et al. [58]	- 69 BPD (mean age ≈ 26.1) - 22 non-BPD Axis II controls	- Routine 16-channel EEG (eyes closed) + hyperventilation and photic stimulation - Blind ratings of dysrhythmias	- Diagnostic Interview for Borderlines (DIB), DSM-III	- Mild abnormalities in 13% of BPD vs. 9.1% controls - Severe abnormalities in 5.7% of BPD vs. 0% controls - Differences not statistically significant - No linkage to specific BPD symptoms
Schauer et al. [59]	- 19 BPD (mean age ≈ 27.47) - 18 HC	- Simultaneous EEG-fMRI - Gambling task (gain/loss feedback) - Focus on theta (4.4–5.8 Hz) and high-beta (22–29 Hz)	- Barratt Impulsiveness Scale (BIS) - Time-frequency (wavelets), BOLD responses	- BPD showed reduced theta power for loss feedback - fMRI: lower activations in left anterior insula and postcentral gyrus - Theta–fMRI coupling differences in dlPFC (loss vs. gain)
Haaf et al. [60]	- 25 female BPD (mean age ≈ 27.2) - 25 female HC	- EEG during cognitive reappraisal task (neg. vs. neutral IAPS) - Theta band (3.5–8.5 Hz) power and connectivity (eLORETA, MIM)	- Emotion ratings and Emotion Regulation Questionnaire (ERQ)	- Both groups reduced negative affect with reappraisal - BPD showed smaller increases in frontal theta and weaker connectivity - Theta power correlated with reappraisal scores in BPD
Vukojević et al. [61]	- 34 MDD - 34 BPD + MDD (ICD-10) - Total 146 EEGs (various conditions)	- 19-channel EEG - Resting, photostimulation, hyperventilation - Machine learning on linear and non-linear features	- Multiple classifiers: SVM, Random Forest, etc.	- No EEG feature set reliably differentiated MDD from BPD + MDD - Overlap in electrophysiological profiles of both groups
Stead et al. [62]	- 64 adolescents (62.5% female, Mage ≈ 14.45) - Varying BPD symptoms (including subclinical)	- 3 min eyes open + 3-min eyes closed resting EEG - Frontal alpha asymmetry (FAA = ln(F4) − ln(F3))	- Cyberball social rejection paradigm - Rejection sensitivity measures	- FAA moderated BPD symptoms and rejection sensitivity - Greater left FAA + high BPD = highest rejection sensitivity - Right FAA associated with moderate, stable levels of rejection sensitivity

Abbreviations: BPD: Borderline Personality Disorder, MDD: Major Depressive Disorder, BD: Bipolar Disorder, OCD: Obsessive–Compulsive Disorder, HC: Healthy Control(s), EC: Eyes Closed; EO: Eyes Open, FEA/FAA: Frontal EEG Asymmetry/Frontal Alpha Asymmetry, PSD: Power Spectral Density, ICA: Integrated Cognitive Assessment or Independent Component Analysis (depending on context), IAPS: International Affective Picture System, DMN: Default Mode Network.

**Table 2:** Table with the assessment of the quality of evidence and the risk of bias in the included studies.

Risk of Bias					Quality of Evidence	Study
Reporting Bias	Attrition Bias	Detection Bias	Performance Bias	Selection Bias		
Low	Low	Low/moderate	Low	Moderate	Moderate	Beeney et al. [39]
Moderate	Low	Low/moderate	Low risk	Moderate/high risk	Moderate	Yun et al. [40]
Moderate	Low	Low/moderate	Moderate	High	Low to moderate	Popkirov et al. [41]
Low/moderate	Low	Low	Low/moderate	Moderate	Low to moderate	Flasbeck et al. [42]
Moderate	Low	Moderate	Low/moderate	High	Low to moderate	Snyder et al. [43]
Moderate	Low	Low/moderate	Low	Moderate	Low to moderate	Deiber et al. [44]
Low/moderate	Moderate	Moderate	High	Moderate	Low to moderate	Russ et al. [45]
Moderate	Low	Moderate	Low/moderate	High	Low to moderate	Arikan et al. [46]
Moderate	Low	Low/moderate	Moderate	High	Low	Ogiso et al. [47]
Low/moderate	Low	Low/moderate	Moderate	Low/moderate	Moderate to high	Kramer et al. [48]
Moderate	Low	Low	Low	Low/moderate	Moderate	Schauer et al. [49]
Low/moderate	Low	Low	Low	Low/moderate	Moderate	Shankar et al. [50]
Moderate	Low	Moderate	Low/moderate	High	Low to moderate	Rezaei et al. [51]
Low/moderate	Low	Low/moderate	Moderate	Moderate/high	Low to moderate	Hegerl et al. [52]
Low/moderate	Low	Low	Low/moderate	Moderate/high	Moderate	Stewart et al. [53]
Moderate	Low	Low	Low/moderate	High	Low to moderate	Nazari et al. [54]
Moderate	Low	Low/moderate	Moderate	High	Low to moderate	Cowdry et al. [55]
Moderate/high	Low	Moderate	Low/moderate	High	Low	Pop-Jordanova et al. [56]
Moderate	Low	Low	Low	Low/moderate	Moderate	De la Fuente et al. [57]
Low/moderate	Low	Low/moderate	Low	Moderate	Low to moderate	Cornelius et al. [58]
Moderate	Low	Low	Low	Low/moderate	Moderate	Schauer et al. [59]
Low/moderate	Low	Moderate	Low/moderate	Moderate	Moderate	Haaf et al. [60]
Moderate/high	Low	Moderate	Low/moderate	High	Low to moderate	Vukojević et al. [61]
Unclear	Low	Moderate	Moderate	High	Low to moderate	Stead et al. [62]

## Data Availability

No new data were created or analyzed in this study. Data sharing is not applicable to this article.
